# A joint optimization QSAR model of fathead minnow acute toxicity based on a radial basis function neural network and its consensus modeling†

**DOI:** 10.1039/d0ra02701d

**Published:** 2020-06-04

**Authors:** Yukun Wang, Xuebo Chen

**Affiliations:** School of Chemical Engineering, University of Science and Technology Liaoning No. 185, Qianshan Anshan 114051 Liaoning China wyk410@163.com; School of Electronic and Information Engineering, University of Science and Technology Liaoning No. 185, Qianshan Anshan 114051 Liaoning China xuebochen@126.com +864125928367

## Abstract

Acute toxicity of the fathead minnow (*Pimephales promelas*) is an important indicator to evaluate the hazards and risks of compounds in aquatic environments. The aim of our study is to explore the predictive power of the quantitative structure–activity relationship (QSAR) model based on a radial basis function (RBF) neural network with the joint optimization method to study the acute toxicity mechanism, and to develop a potential acute toxicity prediction model, for fathead minnow. To ensure the symmetry and fairness of the data splitting and to generate multiple chemically diverse training and validation sets, we used a self-organizing mapping (SOM) neural network to split the modeling dataset (containing 955 compounds) characterized by PaDEL-descriptors. After preliminary selection of descriptors *via* the mean decrease impurity method, a hybrid quantum particle swarm optimization (HQPSO) algorithm was used to jointly optimize the parameters of RBF and select the key descriptors. We established 20 RBF-based QSAR models, and the statistical results showed that the 10-fold cross-validation results (*R*_cv10_^2^) and the adjusted coefficients of determination (*R*_adj_^2^) were all great than 0.7 and 0.8, respectively. The *Q*_ext_^2^ of these models was between 0.6480 and 0.7317, and the *R*_ext_^2^ was between 0.6563 and 0.7318. Combined with the frequency and importance of the descriptors used in RBF-based models, and the correlation between the descriptors and acute toxicity, we concluded that the water distribution coefficient, molar refractivity, and first ionization potential are important factors affecting the acute toxicity of fathead minnow. A consensus QSAR model with RBF-based models was established; this model showed good performance with *R*^2^ = 0.9118, *R*_cv10_^2^ = 0.7632, and *Q*_ext_^2^ = 0.7430. A frequency weighted and distance (FWD)-based application domain (AD) definition method was proposed, and the outliers were analyzed carefully. Compared with previous studies the method proposed in this paper has obvious advantages and its robustness and external predictive power are also better than Xgboost-based model. It is an effective QSAR modeling method.

## Abstract 2

Acute toxicity of the fathead minnow (*Pimephales promelas*) is an important indicator to evaluate the hazards and risks of compounds in aquatic environments.

## Graphical abstract

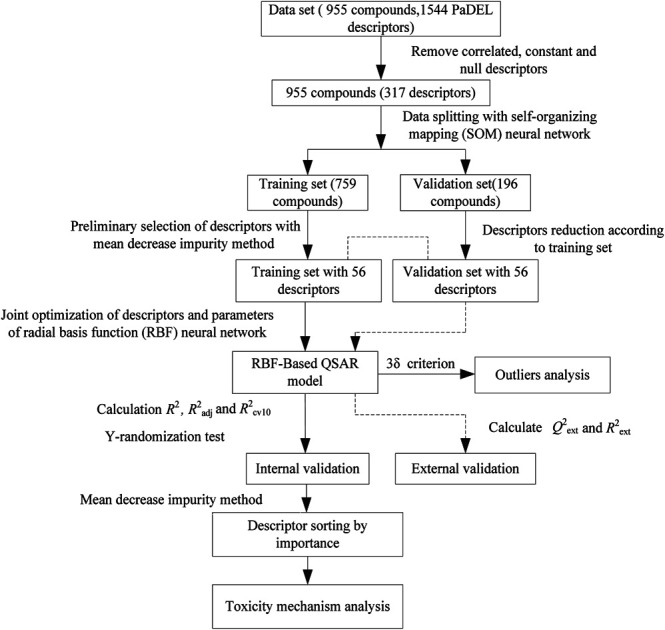

## 1. Introduction

Toxic chemicals may be damaging to the environment and human health.^1^ With the globalization of industrial development, more and more new compounds are being synthesized and can easily enter the water environment *via* industrial production and human actions. These compounds can contaminate the water and become toxicants for aquatic species or other living beings *via* the trophic chain.^2^ The risk assessment of compounds in the aquatic environment has been an increasing focus of governments. Toxicity measurement experiments based on animals are accurate but expensive, time-consuming, and ethically questionable. Quantitative structure–activity relationship (QSAR) models are ideal alternatives because of their higher efficiency and lower cost. In the field of aquatic toxicology, QSARs have been developed as scientifically credible models for predicting the toxicity of chemicals when little or no empirical data are available.^3^ The fathead minnow (*Pimephales promelas*) is important as a biological model in aquatic toxicology studies as it represents one top trophic level of the aquatic food chain.^4^ In previous studies,^2–19^ the acute toxicity to the fathead minnow has become an important indicator to evaluate the hazards and risks of compounds in the aquatic environment. The published QSAR models for the acute toxicity of fathead minnow can mainly be divided into three types: (1) local models based on modes of acute toxic action,^3–7^ (2) global models based on non-congeneric compounds,^2–5,7–13,15–19^ and (3) consensus models composed of multiple global or local models.^4,7,14^

The advantage of local models is that they usually have better statistical performance and offer a better interpretation of the mechanism. The disadvantage of local models is that, for newly synthesized compounds, the toxic mode of action (MOA) may be unknown, which creates difficulties in the application of the models.^5^ Netzeva *et al.* reviewed various local fathead minnow (Q)SAR models based on MOA and analyzed the advantages and disadvantages of local models.^4^ Russom *et al.* proposed a QSAR model that relates the modes of acute toxic action in fathead minnow to chemical structures and properties.^3^ Approximately 600 chemicals were classified as narcotics (three distinct groups), oxidative phosphorylation uncouplers, respiratory inhibitors, electrophiles/proelectrophiles, acetylcholinesterase inhibitors, or central nervous system seizure agents. In the literature,^5^ Wu *et al.* divided the toxicity data (contains 963 compounds) into two groups according to anesthetic toxicity and excess toxicity and obtained two local prediction models of high quality. In the narcosis toxicity model, *R*_adj_^2^ = 0.762 (*R*_adj_^2^ is the adjusted square correlation coefficients of fitting), *R*_cv_^2^ = 0.758 (*R*_cv_^2^ is the correlation coefficients of fitting in cross validation), and *Q*_ext_^2^ = 0.798 (*Q*_ext_^2^ is the square correlation coefficients for external validation set), and, in the excess toxicity model, *R*_adj_^2^ = 0.850, *R*_cv_^2^ = 0.841, and *Q*_ext_^2^ = 0.752. In the study of Yuan *et al.*,^6^ MOA-based local QSAR models were established by partial least squares (PLS) regression for each subset with a single MOA, such as narcosis I, narcosis II, or reactive. The performance of the MOA-based model was performance than that of the global model established in the literature.^6^ Lozano *et al.* studied a consensus QSAR related to global or MOA models and applied those models to acute toxicity for fish.^7^ Although the local model based on MOA showed better performance than that of global models, the authors also noted that the identification of chemical categories may be problematic because chemicals usually show different chemical components, thereby confusing efforts to achieve meaningful classification.

As mentioned above, the development of local models based on MOA is limited by their own characteristics. The global modeling method is still a common method used to establish QSAR models. Global models do not consider the MOA of the compounds, making them easy to establish and apply. So far, many global predictive models for fathead minnow have been published.^1–5,7–13,15–19^ In these studies, a variety of algorithms, such as Random Forest (RF),^1^ Multiple Linear Regression (MLR),^2,5^ a Support Vector Machine (SVM),^9^ and an Artificial Neural Network (ANN)^15^ were used to develop the models. Although some models achieved good statistical results in some aspects of model performance (*R*^2^ = 0.89–0.99 (*R*^2^ is the square correlation coefficients of fitting)^15^ and the best value of *Q*_ext_^2^ of models established in ref. 16 was 0.77), there were still some shortcomings. Some models lacked a validation set or had a small validation set,^15,18^ some models were not cross-verified,^15,16^ and some models lacked application domain (AD).^15,19^

In the study of Wu *et al.*,^5^ a prediction model of the acute toxicity to fathead minnow with 963 compounds was established; this model combined the genetic algorithm (GA) and MLR. This model strictly follows OECD principles, and its prediction accuracy is the best currently obtained with such a big data set. The performance of the model is *R*_adj_^2^ = 0.701, *R*_cv_^2^ = 0.700, and *Q*_ext_^2^ = 0.641 (less than 0.7). Although the model's fitting ability and robustness meet the requirements of the QSAR model, the external predictive power of the model is still poor. The main reason for the poor external predictive power of the MLR model is the complex nonlinearity between its descriptors and acute toxicity. MLR lacks a nonlinear fitting ability, which limits improvements to the model's performance. We found consensus modeling of fathead minnow acute toxicity in this literature.^4,7,14^ Consensus modeling is a potential method to improve the external prediction ability of these QSAR models.

Our study uses a data set (contains 955 molecules) and simpler 0–2D PaDEL-descriptors^20^ to establish a QSAR model with better fitting ability, robustness, and external predictive power, as well as a wide AD. Meanwhile, the mechanism of acute toxicity and causes of outliers are explained reasonably.

In previous studies,^5,9,10,15,21–26^ machine learning methods, such as SVM^9,10,22^ and ANN,^10,15,23,25^ and intelligent optimization algorithms, such as GA,^5,21,26^ ant colony optimization algorithm (ACO),^24^ and particle swarm optimization (PSO),^22^ have been widely used in QSAR modeling. The nonlinear fitting ability of the machine learning algorithm and the powerful optimization ability of the intelligent optimization algorithm significantly improved the performance of the QSAR model. In our study, we employ an RBF neural network and a hybrid quantum particle swarm optimization (HQPSO) algorithm^27^ to capture the nonlinear relationship between the molecular structure and acute toxicity in fathead minnow. The RBF neural network has a strong nonlinear fitting ability and has been successfully applied to different QSAR problems. The HQPSO algorithm is an improved quantum particle swarm optimization algorithm proposed by our research team, which has successfully solved the complex problem of Au cluster structure optimization.^27^

In the QSAR modeling process, the selection of the descriptor and parameters of the QSAR model belong to discrete optimization and continuous optimization problems, respectively. A joint optimization of the descriptors and model parameters is difficult to implement. Although the “Selecting descriptors first, and then modeling” method is usually used to establish a QSAR model, it remains difficult to guarantee that both descriptors and model parameters will be optimized at the same time. This limit further improvement of the QSAR model's performance. In this paper, the HQPSO algorithm with a new fitness function and a new parameter encoding method were employed to jointly optimize the molecular descriptors and the QSAR model parameters to ensure that the most appropriate molecular descriptors were selected under the optimal model parameters. To our knowledge, this jointly optimized method has never before been used in QSAR modeling for the acute toxicity prediction of fathead minnow.

Through the primary selection of descriptors and joint optimization of the model parameters and descriptors, we established 20 RBF-based models. Under the established QSAR models, we evaluated the importance of the descriptors by the mean decrease impurity method^28^ and concluded that the water distribution coefficient, molar refractivity, and first ionization potential are important factors affecting the acute toxicity of fathead minnow. After that, we established a consensus model to improve the external predictive power of RBF-based models. The statistical results showed that the performance of the consensus model was greatly improved. Finally, a frequency-weighted and distance (FWD)-based AD definition method was proposed, and the outliers were analyzed carefully.

## 2. Data acquisition and computing resources

### 2.1. Data acquisition

In this paper, the fathead minnow acute toxicity data were obtained from the literature.^5^ In ref. 5, these data have been rigorously screened, and the outliers have been eliminated. The data set contains 963 organic compounds, including aldehyde, carboxylic acid, esters, amines, alkanes, alkenes, alkyne, alcohols, nitrobenzene, halohydrocarbon, ketones, phenols, ethers, nitriles, and heterocycles. In these data, −log LC_50_ (mol l^−1^) (LC_50_ is the 96 h 50% lethal concentration) was chosen as the endpoint. After data collection, we used CAS code for each compound to query its SMILES code^29^ and then used these SMILES codes to calculate the descriptors of compounds online through the PaDEL software.^30^ To construct a more stable QSAR model, we only calculated 1544 0–2D descriptors to avoid the uncertainty caused by molecular structure optimization when calculating 3D descriptors. In the collected data set, 8 compounds cannot calculate their descriptors, among which the CAS codes of seven may have errors, and their SMILES can't be queried; the SMILES of the other one cannot be used to calculate descriptors using the PaDEL software. A detailed information of the deleted compounds is listed in Table S1 in the ESI.† Finally, we obtained a modeling data set containing 955 compounds.

### 2.2. Computing resources

In the QSAR modeling process, the program code of the data splitting, SOM neural network, HQPSO algorithm, internal and external validation, AD, outlier analysis, RBF-based QSAR model and its consensus model in this paper were completed using the MATLAB 2014 software (MathWorks, Natick, Massachusetts, USA). PyCharm 2020.1(Contain python3.7) was used to implement Xgboost-based model. The computer's operating system was WIN10.

## 3. RBF-based QSAR modeling with the joint optimization method

### 3.1. The general idea of the modeling process

From data acquisition, data segmentation, descriptor selection, and model parameter optimization to performance evaluation, the QSAR model we established was completely under the constraints of the OECD principle.

In this paper, a total of 955 compounds (1544 descriptors) were prepared for our QSAR model. For the first approach, descriptors with constant or null values were excluded. In addition, if the descriptors were found to be correlated pairwise (greater than 0.85), then the descriptor that has the least correlation with acute toxicity (−log LC_50_) was excluded in a preliminary step to reduce redundancy. In total, 317 PaDEL descriptors were reserved and used to develop the QSAR models. The dataset with 317 descriptors is listed in the ESI (Data_317Descriptors and Acute Toxicity.xls).†

**Fig. 1:**
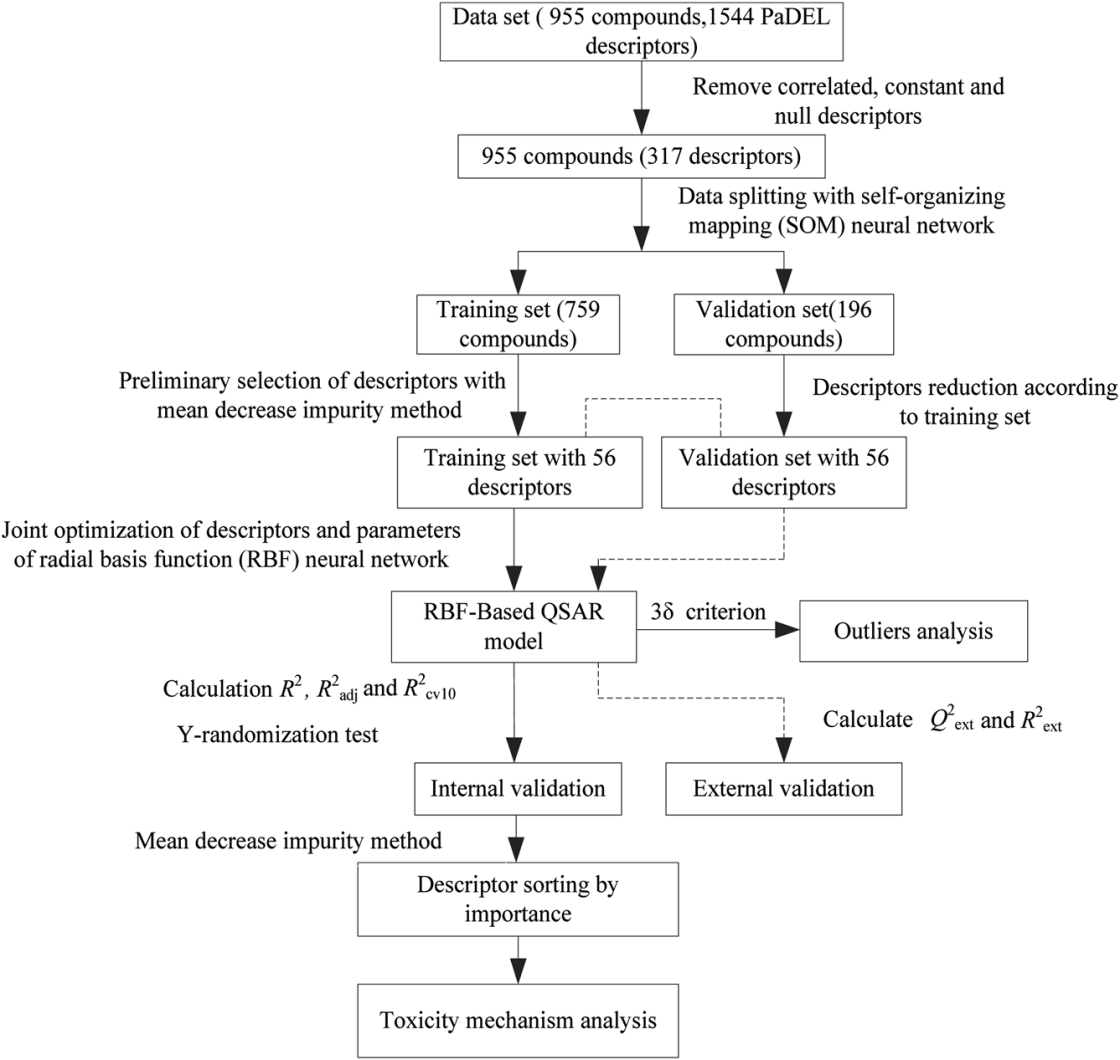
Flowchart of the radial basis function (RBF)-based method.

Next, the modeling data set was split into training and validation sets using the self-organizing mapping (SOM) neural network,^31^ and the preliminary selection of descriptors based on the mean decrease impurity method was implemented to simplify the model structure. To improve the performance of the model, we employed the HQPSO algorithm to jointly optimize the descriptors and parameters of the RBF-based QSAR model. Lastly, internal validation, external validation, AD analysis, and outlier (compounds with a large forecast deviation) analysis were implemented. The RBF-based QSAR model-building workflow is shown in Fig. 1.

### 3.2. Splitting the modeling data set into training and validation sets

In the OECD principle, external validation is the only way to confirm the true predictive power of a QSAR model. The real predictive power of a QSAR model must be characterized by the predictive accuracy of the activity of the compounds not used in model development. This type of assessment requires the use of an external validation set. It can be seen from previous studies^31,32^ that the predictive power of a QSAR model for different structural compounds will be better if diverse training data are obtained.

The data splitting method proposed in this paper is an improvement of literature.^5^ In literature,^5^ they sorted the data in ascending order according to their acute toxicity values (−log LC50) and picked one out of every five to constitute the validation set. They only considered the diversity and uniformity of toxicity values in training and validation set, but not the structural diversity of compounds.

To ensure the diversity of the training and validation sets from structures and toxicity values, we introduced the idea of “clustering first and then classifying”.

Firstly, we used the SOM neural network to divide the data set into several groups and each group of data had structural similarity. Self organizing maps (SOM), a self organizing map neural network, can be used for unsupervised learning clustering of data. It's a kind of neural network with only input layer and hidden layer. A node in the hidden layer represents a class that needs to be aggregated. During the training, the method of “competitive learning” is adopted. Each input sample finds a node that best matches it in the hidden layer, which is called its activation node, or “winning neuron”. Then the parameters of the active node are updated by the gradient descent method. At the same time, the points close to the active node update the parameters according to their distance from the active node.^33^ At present, it is widely used in data clustering analysis. The hidden layer of SOM with 9 nodes was selected to divide the compounds into 9 or less than 9 groups according to their structural similarity. The number of groups is selected according to our experience. If the selected groups are too small, even if, there is only one group, it tends to select training and validation sets according to literature.^5^ If there are too many groups, the selection tends to randomly select training and validation sets.

**Fig. 2:**
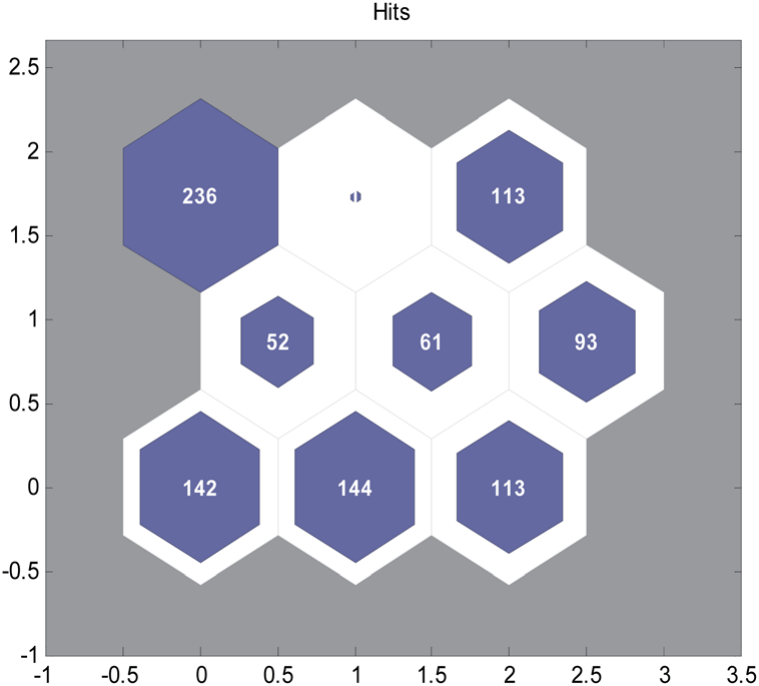
Clustering results of the self-organizing mapping (SOM) neural network.

The SOM neural network was generated by the MATLAB toolbox, including 317 inputs and 9 nodes in hidden layer. The clustering results of the modeling data after 300 iterations of training are shown in Fig. 2.

Then, for each group of data, we sorted the data in ascending order according to their acute toxicity values (−log LC_50_) and picked one out of every five to constitute the validation set. Finally, we obtained a training set containing 759 compounds and a validation set containing 196 compounds. A detailed classification is listed in the ESI (Data splitting to Training and Validation Set.xls).†

Although random selection may also be able to achieve the structural diversity of training and validation compounds, it can't guarantee the uniformity distribution of toxicity values.

**Fig. 3:**
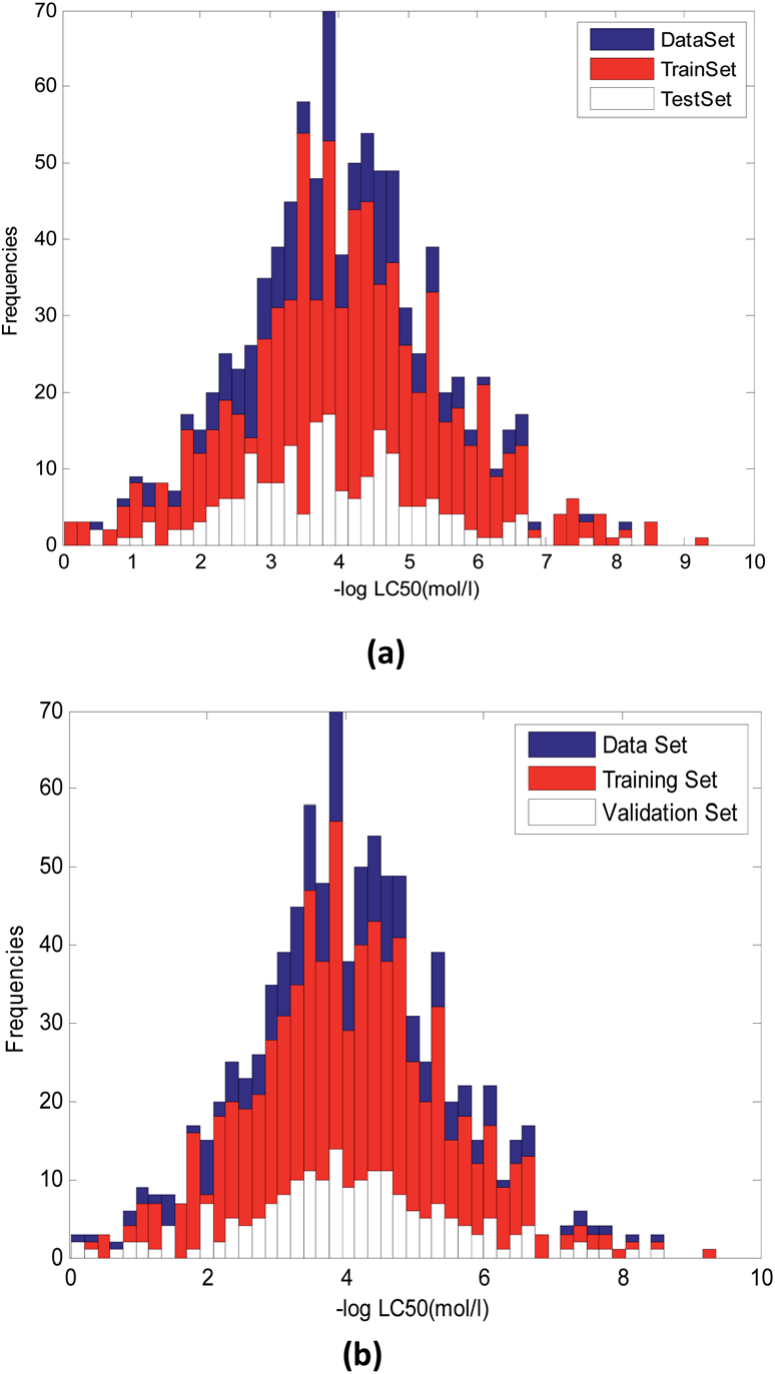
Distribution of compounds with various toxicity values in training and validation set. (a) Random method. (b) Method proposed in this paper.

It can be seen from Fig. 3, the random method and method used in this paper has some difference. Fig. 3(a) used random method and Fig. 3(b) used the proposed method. We can see that in Fig. 3(a), the uniformity of compounds with various toxicity values in training and validation sets was worse than that in Fig. 3(b)

**Fig. 4:**
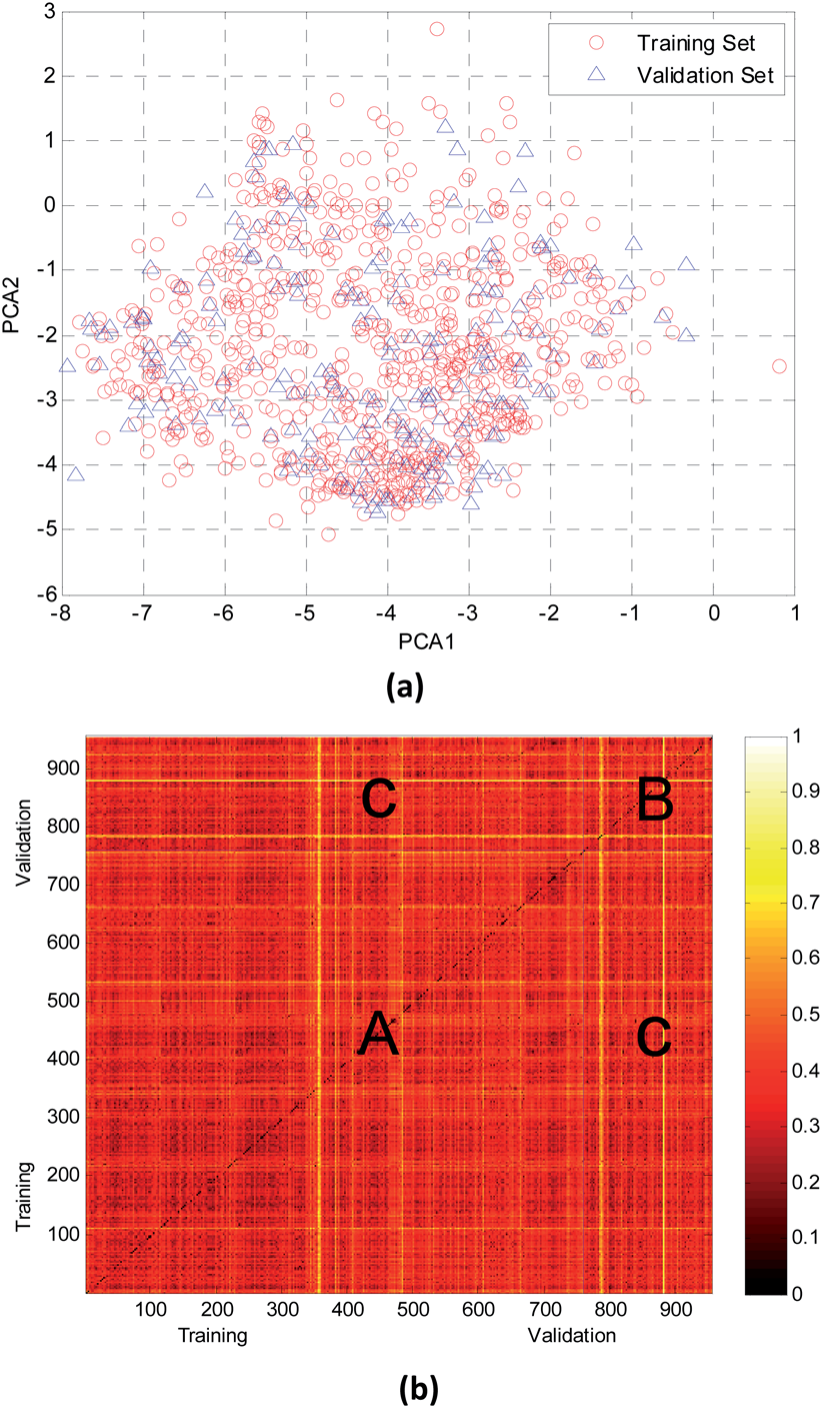
Chemical diversity distribution of the training and validation sets. (a) The chemical space was analyzed using principal component analysis (PCA) method. (b) A heat map of molecular similarity plotted by normalized Euclidian distance metrics for the training and validation sets.

In this study, the chemical space distribution was analyzed using principal component analysis (PCA).^34^ As shown in the PCA plot of the compounds based on the 317 selected molecular descriptors (Fig. 4(a)), the compounds in the validation set were basically distributed within the chemical space of the training set. The Euclidian distance metrics of the two datasets were calculated using 317 PaDEL descriptors to further evaluate the chemical diversity of the compounds. A larger Euclidian distance metric means a more diverse data set.^35^ The training and validation sets were compared with each other, and the heat map of the normalized Euclidian distance metrics is shown in Fig. 4(b).

As can be seen from Fig. 4(b), the colors of regions A and B are similar with each other. It is obvious that the diversity of training and validation sets are similar. The color between the training set and validation set (region C, which has only a few bright lines) also illustrated that most compounds in the training and validation sets shared a similar chemical space.

Therefore, on the one hand, compared with the methods in literature,^5^ the training and validation set produced by our method may have more structural diversity. On the other hand, compared with the random method, the toxicity distribution of the compounds in the training and validation sets was more uniform. So, we think our method can improve the effect of data splitting to a certain extent.

### 3.3. Preliminary selection of descriptors

In QSARs, using fewer descriptors helps to avoid over-fitting and to establish meaningful models whose chemical mechanisms are easy to explain. At the same time, deleting the unimportant descriptors will reduce the computational complexity of the joint optimization algorithm. We implemented the importance evaluation and preliminary selection of descriptors based on the mean decrease impurity method with the RBF neural network.

The RBF neural network consists of three layers, each of which has a completely different role. The input layer is composed of some perceptual units, which connect the network with the external environment; the second layer is the only hidden layer in the network, whose function is to make nonlinear transformation from the input space to the hidden layer space. In most cases, the hidden layer space has a higher dimension; the output layer is linear, which provides response for the activation mode acting on the input layer.

**Fig. 5:**
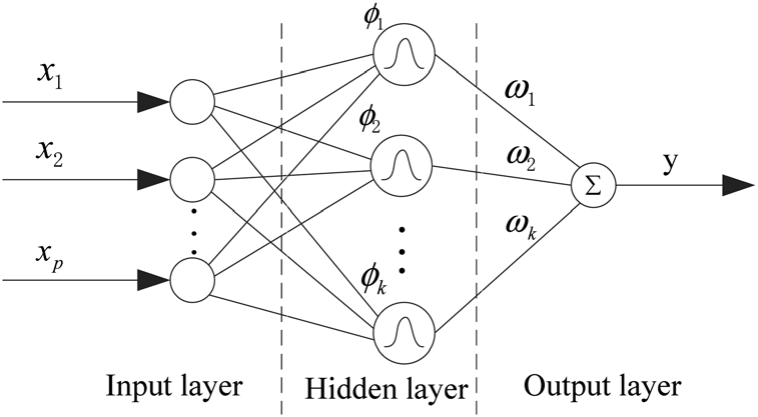
Structure of the RBF neural network.

It has a powerful nonlinear fitting ability and a fast training speed. The structure of a network with *p* inputs, *k* hidden nodes, and 1 output is shown in Fig. 5.

The output of the network is as follows:1
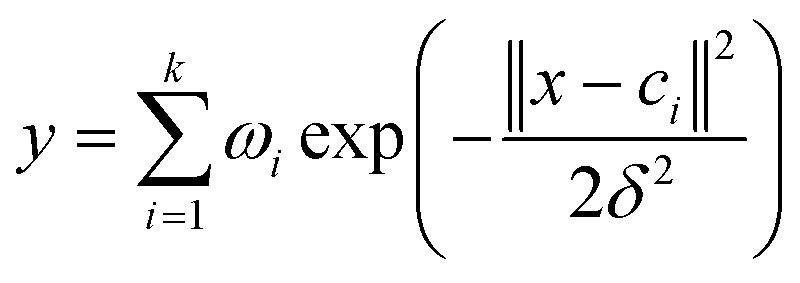
where *x* is a *p*-dimensional input vector, and *c*_*i*_ is the center vector of the *i*th hidden layer node, *δ* is the spread of the radial basis function (activation function), and *ω*_*i*_ is the weight from the *i*th hidden layer node to the output node.^36^ The parameters affecting the performance of the RBF model are *δ* and *ε* (the mean squared error of the experimental and calculated responses of the training object). The smaller the value of *ε* is, the stronger the fitting ability of the trained model will be. But too small *ε* will lead to over fitting of the model. *δ* and *ε* affect the fitting and generalization ability of the model, respectively.^36^

The implementation steps of mean decrease impurity method are as follows:

Step 1: Dividing the training set, we randomly obtained two parts: Data A and Data B. Data A contains 90% of the training set, and Data B contains the other 10%.

Step 2: We constructed an RBF model with 317 inputs (317 descriptors) and 1 output (−log LC_50_) using Data A.

Step 3: Using data B to test the accuracy of the model, we calculated *R*_0_^2^, *R*_0_^2^ is written in eqn (2).2
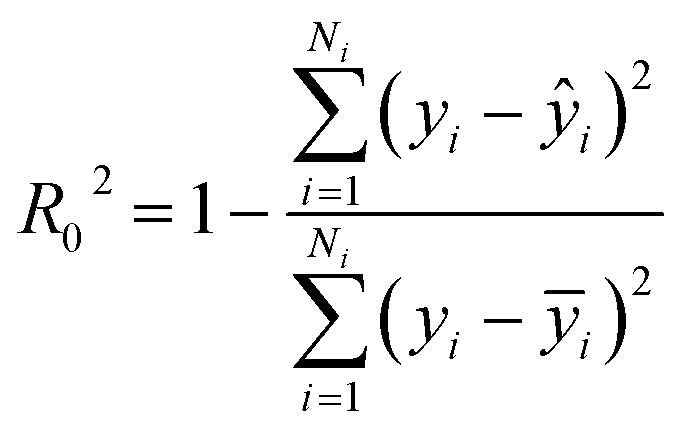
where *ȳ* is the average response of the testing objects, while *y*_*i*_ and *ŷ*_*i*_ are the experimental and predicted responses of the *i*th testing object, respectively. *N*_*i*_ is the number of test samples in the *i*th model.

Step 4: We randomly shuffled the descriptor values of each column of data B in turn (only one column is shuffled at a time) to form new data sets B_1_(*i*), (*i* = 1, 2, …, 317). The prediction accuracy of the RBF model against the 317 data sets was tested and recorded as *R*_1_^2^(*i*), (*i* = 1, 2, …, 317).

Step 5: We calculated the decrease in the impurity value of each descriptor, recorded as Ir(*i*). Ir(*i*) is defined as eqn (3).3
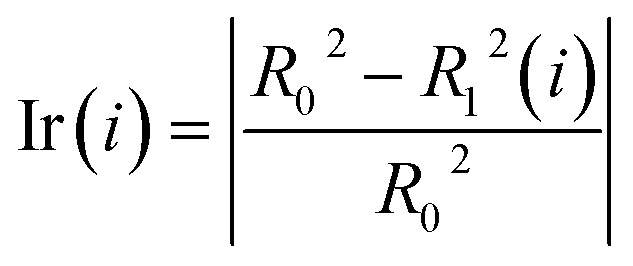


Step 6: We repeated step 1 to step 5 50 times, and then the value of the mean decrease impurity (
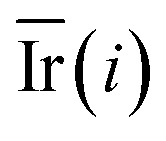
) was calculated. The importance of each descriptor was evaluated by 
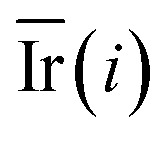
.

When using the above method to evaluate the importance of descriptors, the maximum number of neurons in the RBF neural network was set to 500 (greater than the number of descriptors). There are too many descriptors, so computing power cannot meet the requirements of the joint optimization of RBF neural networks and descriptors. To avoid the influence of RBF neural network parameters on the importance evaluation of the descriptors, we randomly selected 100 sets of different parameters for the RBF neural network (*ε* ∈ [10^−3^, 10^−1^] and *δ* ∈ 1, 5) and repeated the above evaluation method 100 times.

**Fig. 6:**
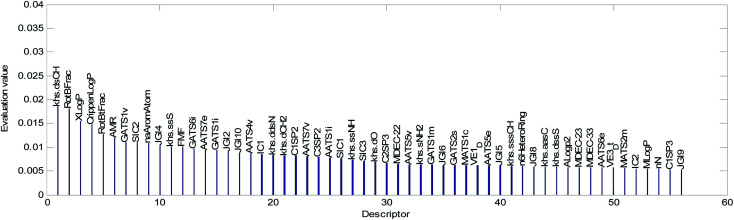
Descriptor importance ranking and the results of the descriptor selection.

The mean value of the 100 evaluation results was used to judge the importance of each descriptor. In our study, descriptors with an evaluation value greater than 0.01 were retained and graphically shown in Fig. 6. Finally, the 56 descriptors shown in Fig. 6 were selected to establish our QSAR model. The results of the descriptor preliminary selection and detailed information on the 56 selected descriptors are listed in the ESI (Preliminary selection of 56 Descriptors.xls and Table S2).†

### 3.4. Establishment of joint optimization method

In essence, the mean decrease impurity method is a single factor analysis method. Although the number of descriptors is reduced, the selected descriptors may still have multicollinearity, which will affect the performance of the QSAR model.

Only the joint optimization of the descriptors and model parameters can guarantee that the most appropriate molecular descriptors are selected under the optimal model parameters to improve the performance of the QSAR model. In this paper, HQPSO algorithm was used to jointly optimize the hyper parameters and input variables (descriptors) of RBF model.

HQPSO is a variant of Quantum-behaved particle swarm optimization (QPSO). QPSO is an improved particle swarm optimization (PSO) algorithm with quantum behavior, it was proposed in 2004 by Sun.^37^ According to the basic principle of quantum mechanics, QPSO uses wave function instead of position and velocity to describe the state of particles. It has many advantages, such as few parameters, simple operation and strong convergence ability. However, for complex multi-modal optimization problems, similar to other algorithms, the QPSO algorithm still suffers from premature and poor precision. The HQPSO algorithm was proposed to enhance diversity of the population, balance the exploration and exploitation abilities and improve the precision of QPSO algorithm. In HQPSO, new global, local and enhanced search strategy, Lévy flight and hopping operation technology, and new convergence speed control method were introduced. Combined with the advantages of quantum behavior of QPSO and hybrid operation strategy, the performance of HQPSO algorithm had been improved. The numerical test results had demonstrated that HQPSO is of better performance than PSO, QPSO and some other comparison algorithms. This algorithm has been successfully applied to the optimization of ground state structure of Au_*n*_ (*n* = 12–30) cluster in chemistry (a typical NP problem).

In this paper, we focused on the application of HQPSO algorithm. The detailed implementation steps and mathematical equation of HQPSO algorithm can be found in literature.^27^

The selection of the descriptors and the optimization of the parameters of the QSAR model belong to discrete and continuous optimization problems, respectively. In intelligent optimization algorithms, it is difficult to solve the joint optimization problem with traditional binary coding or real coding methods. In this paper, we propose a new coding method, which achieves the coding of descriptors in two steps; this process uses real coding and binary decoding. In the HQPSO algorithm, we adopt a real coding strategy. To realize the discrete optimization of the descriptors, we need to transform the real code of the descriptors into binary code.

Next, we will explain in detail the encoding methods of the descriptors and the model parameters of the RBF neural network. In our model, 56 descriptors and two parameters (*ε* and *δ*) of the RBF neural network need to be optimized. For the HQPSO algorithm, we define an 8-dimensional initial population. The first two dimensions represent the parameters of the RBF neural network, with a value range [10^−4^, 5], that is, *ε* ∈ [10^−4^, 5], and *δ* ∈ [10^−4^, 5]. The last six dimensions represent the descriptors, with a value range [0, 1023]. In the last six dimensions, each dimension represents 10 descriptors. A value of 0 (expressed in binary as ‘0000000000’) means that no descriptor was selected, 1023 (expressed in binary as ‘1111111111’) means that all descriptors were selected, and 563 (expressed in binary as ‘1000110011’) means that the 1st, 2nd, 5th, 6th, and 10th descriptors were selected.

Here, we assume that the initial solution randomly generated by the HQPSO algorithm is Pop = [0.01, 0.25, 515.6, 511.9, 91.0, 0.15, 400.3, 43.7]. Obviously, this is a real coding strategy. Therefore, we need to do the following to transform the initial solution for the implementation of joint optimization:

(1) Obtain the parameters of the RBF neural network from the 1st and 2nd dimensions of Pop: *ε* = 0.01 and *δ* = 0.25.

(2) For the last six dimensions of Pop, round first and then decode it into binary:

[515.6, 511.9, 91.0, 0.15, 400.3, 43.7] → 0, 44, 91, 400, 512,516 → [1000000100, 1000000000, 0001011011, 0000000000, 0110010000, 0000101100]

From the binary decoding results, we can see that the 3rd, 10th, 20th, 21st, 22nd, 24th, 25th, 27th, 45th, 48th, 49th, 52nd, 53rd, and 55th descriptors were selected as the input variables of the RBF neural network. After decoding, we can use these descriptors and parameters of RBF to establish an RBF-based QSAR model.

RBF neural network has strong fitting ability, but it is easy to over fitting. For an RBF model, the larger the value of *R*_0_^2^, the stronger the fitting ability of the model; the larger the value of *R*_cv10_^2^, the stronger the robustness of the model. Our aim is to establish a robust QSAR model with good fitting ability. During the experiment, we found that when the value of *R*_0_^2^ is too large, which will cause the value of *R*_cv10_^2^ to become smaller; the value of *R*_cv10_^2^ is too large, which also causes the value of *R*_0_^2^ to be smaller (the values of *R*_0_^2^ and *R*_cv10_^2^ can be adjusted by adjusting the parameters of RBF neural network). That is to say, if the fitting ability of the model is too strong, the robustness of the model will be worse, and if the robustness of the model is too strong, the fitting ability of the model will be worse. Only choosing the reciprocal of *R*_0_^2^ or *R*_cv10_^2^ as the fitness can't make the model get good performance. To balance the fitting ability and robustness of the model, the fitness function of the HQPSO algorithm is defined as eqn (4):4
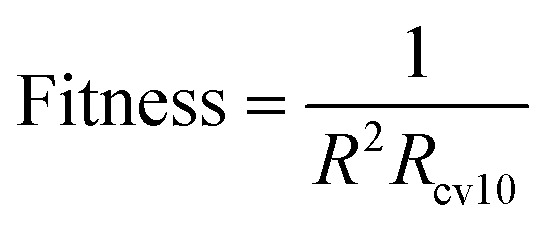


*R* ^2^ and *R*_cv10_^2^ are defined as eqn (5) and (6), respectively.5
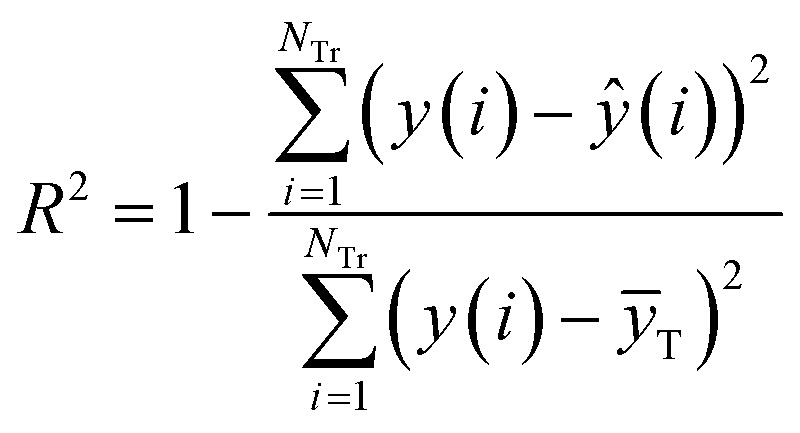
6
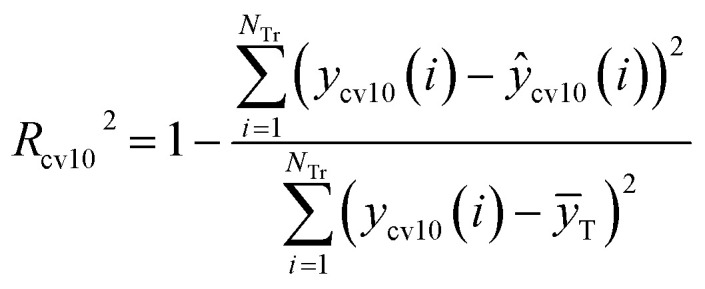
where *N*_Tr_ is the number of compounds in the training set, *ȳ*_T_ is the average response of the training objects, and *y*(*i*) and *ŷ*(*i*) are the experimental and the predicted responses of the *i*th training object, respectively. *y*_cv10_(*i*) and *ŷ*_cv10_(*i*) are, respectively, the experimental and predicted responses of the *i*th training object in 10-fold cross validation. To avoid illegal value of fitness, when the value of *R*^2^ or *R*_cv10_^2^ is not great than 0, we set their value to 0.0001.

**Fig. 7:**
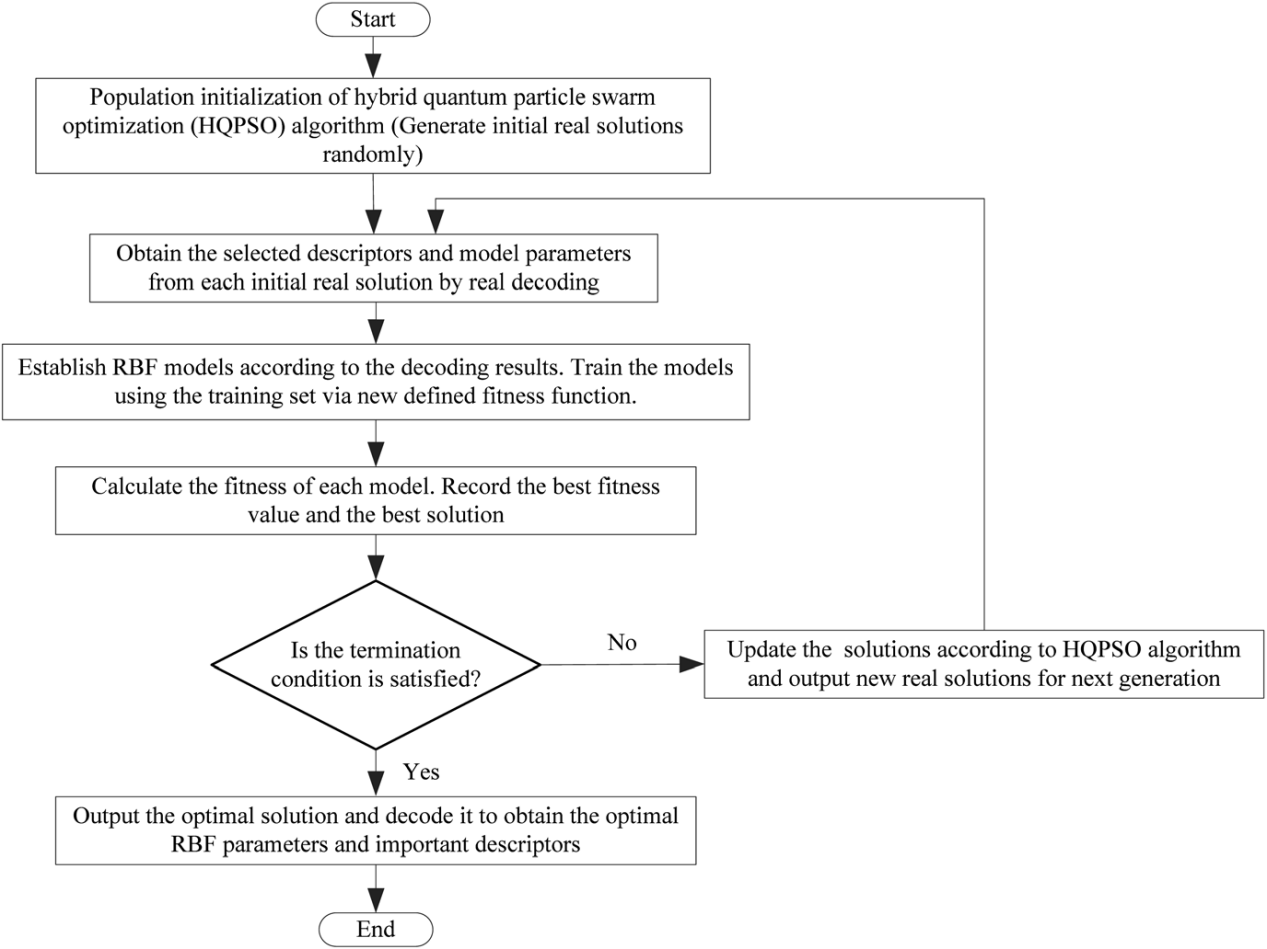
Flow chart of the joint optimization algorithm.

The flow chart for the joint optimization of the QSAR model by the HQPSO algorithm is shown in Fig. 7.

Considering the actual requirements of joint optimization and our computing power, the parameters of the HQPSO algorithm are set as follows: the population size is 30, the number of maximum iterations is 1000, and the internal parameters are *λ* = 1 and *L* = 10 (the values of *λ* and *L* are selected according to ref. 27). To avoid over-fitting, we set the following constraints in the optimization process: 0 < *R*^2^ − *R*_cv10_^2^ < 0.3.

### 3.5. Development and validation of the RBF-based QSAR model

After joint optimization, an optimized RBF-based QSAR model (No. 1) for acute toxicity in fathead minnow was obtained with the training set (containing 759 compounds), and 22 molecular descriptors were selected in the model. The optimized parameters of the RBF neural network were *ε* = 0.0096 and *δ* = 1.1189.

In the OECD principle, QSAR model validation becomes an essential step in developing a statistically valid and predictive model because the real utility of a QSAR model lies in its ability to accurately predict the modeled properties for new compounds. The following two approaches were used to validate the established RBF-based QSAR model:

(1) Internal validation

Decision coefficient: The value of *R*^2^ is 0.9001, and the adjusted decision coefficient *R*_adj_^2^ is 0.8973.

Cross-validation test: The value of the 10-fold cross-validation of *R*_cv10_^2^ is 0.7074. According to the literature,^38^ the cross-validation result must be greater than 0.5 in a robust QSAR model.

Over fitting: *R*^2^ − *R*_cv10_^2^ < 0.3, there is no over-fitting in the model.

*Y*-randomization test: In this test, the dependent-variable vector (*Y*-vector) was randomly shuffled, and a new QSAR model was developed using the original independent variable matrix. It was expected that the resulting QSAR models would have low *R*^2^ and *R*_cv10_^2^ values.^39^ This process was repeated 10 times, and the statistical results for 10 runs are listed in Table 1.

**Table 1:** Statistical results of the *Y*-randomization test

No.	*R* ^2^_yrand	*R*_CV10_^2^_yrand
1	0.0014	−1.0940
2	0.0015	−1.3021
3	0.0014	−1.3484
4	0.0039	−1.1477
5	0.0001	−1.3669
6	0.0011	−1.1941
7	0.0002	−1.3974
8	0.0009	−1.2096
9	0.0004	−1.1710
10	0.0017	−1.5953

Topliss ratio: The QSAR model also fulfills the rule of thumb condition (that is, the topliss ratio), whereby the chemical number over the number of selected variables should be at least 5.^40^

(2) External validation

The external predictive power of the models was tested through the validation set and evaluated by the *R*_ext_^2^ and *Q*_ext_^2^. We calculated *R*_ext_^2^ (using eqn (7)) and *Q*_ext_^2^ (using eqn (8)), and the values were 0.7043 and 0.7041, respectively.

The equation of *R*_ext_^2^ is given as:7
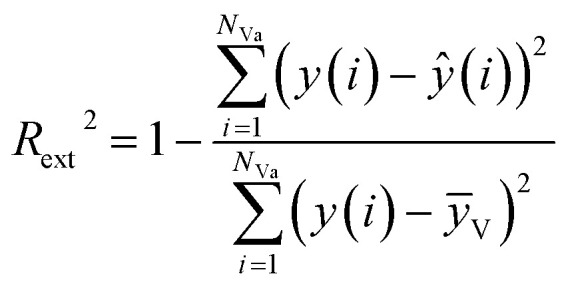
where *N*_Va_ is of the number of compounds in the validation set; *ȳ*_V_ is the average response of the validation objects, while *y*(*i*)and *ŷ*(*i*) are the experimental and predicted responses of the *i*th validation object, respectively.

The equation of *Q*_ext_^2^ is given as:8
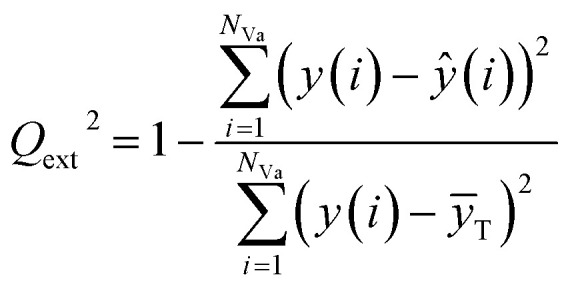
where *N*_Va_ is of the number of compounds in the validation set; *ȳ*_T_ is the average response of the training objects, while *y*(*i*)and *ŷ*(*i*) are the experimental and predicted responses of the *i*th validation object, respectively.

Golbraikh and Tropsha criteria^41^ is also used to evaluate the performance of the RBF-based QSAR model. *k* = 0.9926 (*k*′ = 0.9696) and (
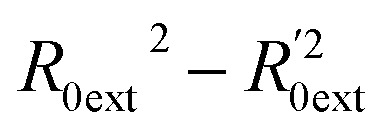
)/*R*_0ext_^2^ = 0.0067. *k* and *k*′ are the corresponding slopes of regression lines through the origin. *R*_0ext_^2^ and 
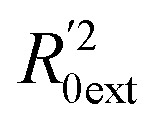
 are calculated forcing the regression line to pass through the origin; for acceptable QSAR predictive models, 0.85 < *k*, *k*′ < 1, 15 and (
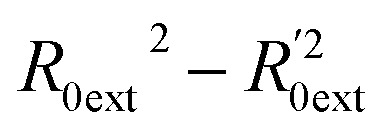
)/*R*_0ext_^2^ < 0.1.^42,43^

Therefore, the RBF-based QSAR model can pass the internal and external validation successfully.

The relatively high quality of *R*_adj_^2^, *R*_ext_^2^ and *Q*_ext_^2^ indicate that the model has a good fitting ability, as well as a good external prediction ability. The high value of *R*_cv10_^2^ in the cross-validation test and the poor values of *R*^2^_yrand and *R*_CV10_^2^_yrand in the *Y*-randomization test ensure the robustness of the model.

**Fig. 8:**
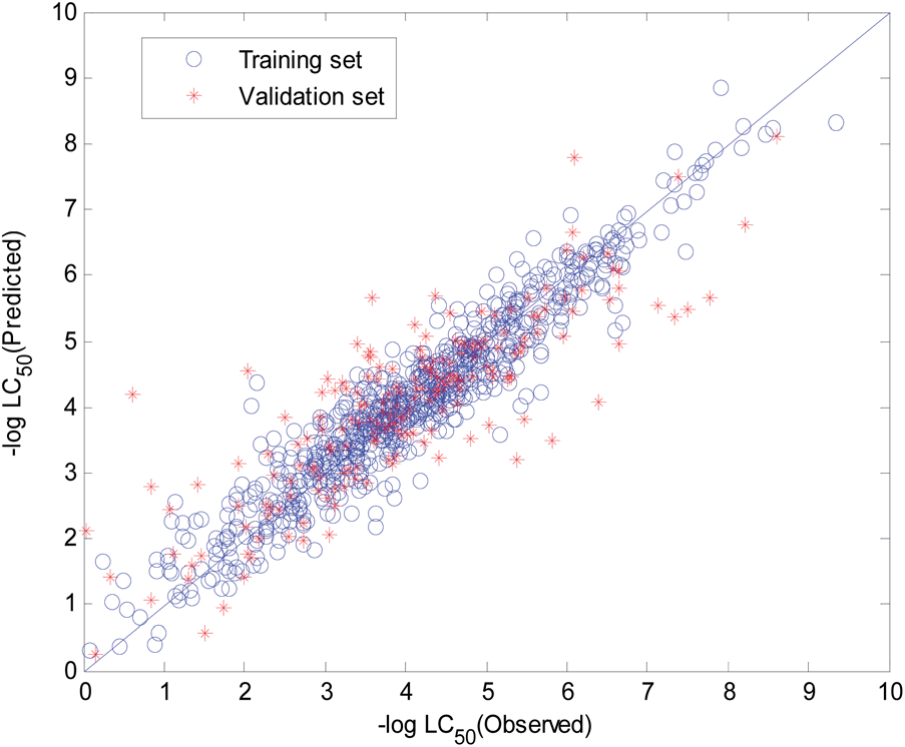
Visual predictive performance of the RBF-based quantitative structure–activity relationship (QSAR) model No. 1.

The visual predictive performance of the RBF-based model is shown in Fig. 8. The blue circles represent compounds in the training set, and the red cross stars represent compounds in the validation set. The solid line shows that the experimental and predicted values are the same. Blue the circles and red cross stars are distributed more-or-less symmetrically on both sides of the solid line, which indicates the good predictive power of the model.

**Fig. 9:**
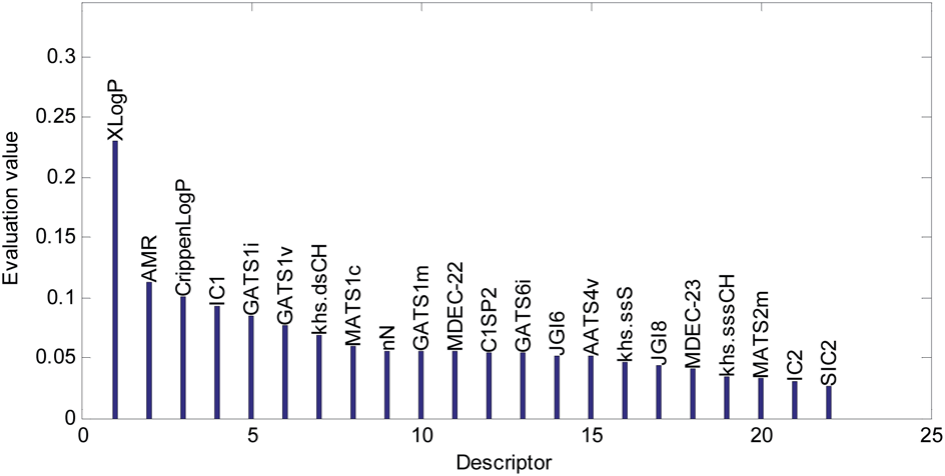
Molecular descriptors selected in QSAR model No. 1.

The descriptors were sorted in descending order according to the importance of the variables and are graphically shown in Fig. 9. The importance of the descriptors was evaluated by the mean decrease impurity method under the optimized RBF neural network.

### 3.6. Stability analysis of the RBF-based models

Intelligent optimization algorithms, such as GA and PSO, have a certain randomness, and the HQPSO algorithm is no exception. Moreover, the joint optimization of the RBF-based QSAR model in this paper is a very complex and time-consuming optimization problem. Within the allowable range of computing resources and time, the solution obtained by each run of the HQPSO algorithm may only offer a different local minima solution, which may affect the stability of the performance of the QSAR model. Therefore, we established another 19 models with the joint optimization algorithm and evaluated the stability of the models. These models also passed internal validation and external validation smoothly. The parameters of the 20 RBF neural networks optimized by the HQPSO algorithm and the performance of the 20 RBF-based QSAR models are shown in Table 2.

**Table 2:** The parameters and performance of the 20 RBF-based QSAR models

No.	Parameters		Performance				
	*ε*	*δ*	*R*_0_^2^	*R*_adj_^2^	*R*_cv10_^2^	*R*_ext_^2^	*Q*_ext_^2^
1	0.0096	1.1189	0.9001	0.8973	0.7074	0.7043	**0.7041**
2	0.0100	1.8090	0.8957	0.8913	0.7026	0.7318	**0.7317**
3	0.0138	1.9633	0.8560	0.8513	0.7163	0.6778	0.6754
4	0.0130	2.2170	0.8643	0.8583	0.7099	0.7281	**0.7257**
5	0.0147	1.5330	0.8473	0.8432	0.7063	0.6756	0.6750
6	0.0129	1.7091	0.8657	0.8604	0.7024	0.6861	0.6846
7	0.0152	1.7533	0.8415	0.8356	0.7214	0.7029	**0.7017**
8	0.0876	1.9164	0.9093	0.9047	0.7008	0.6871	0.6862
9	0.0143	3.6643	0.8526	0.8459	0.7030	0.7284	**0.7267**
10	0.0115	1.5703	0.8809	0.8772	0.7200	0.6590	0.6551
11	0.0066	1.1717	0.9312	0.9290	0.7072	0.7081	**0.7079**
12	0.0136	1.3718	0.8588	0.8539	0.7062	0.6828	0.6820
13	0.0139	1.6444	0.8559	0.8512	0.7189	0.6973	0.6956
14	0.0130	2.4622	0.8639	0.8585	0.7011	0.6921	0.6889
15	0.0134	2.6968	0.8605	0.8553	0.7102	0.6591	0.6480
16	0.0128	2.7330	0.8668	0.8615	0.7347	0.6563	0.6507
17	0.0145	2.8846	0.8484	0.8418	0.7099	0.6815	0.6736
18	0.0110	2.1840	0.8851	0.8802	0.7143	0.6962	0.6879
19	0.0139	2.5180	0.8554	0.8504	0.7066	0.6617	0.6555
20	0.00732	1.9213	0.9237	0.9200	0.7065	0.6684	0.6612

**Fig. 10:**
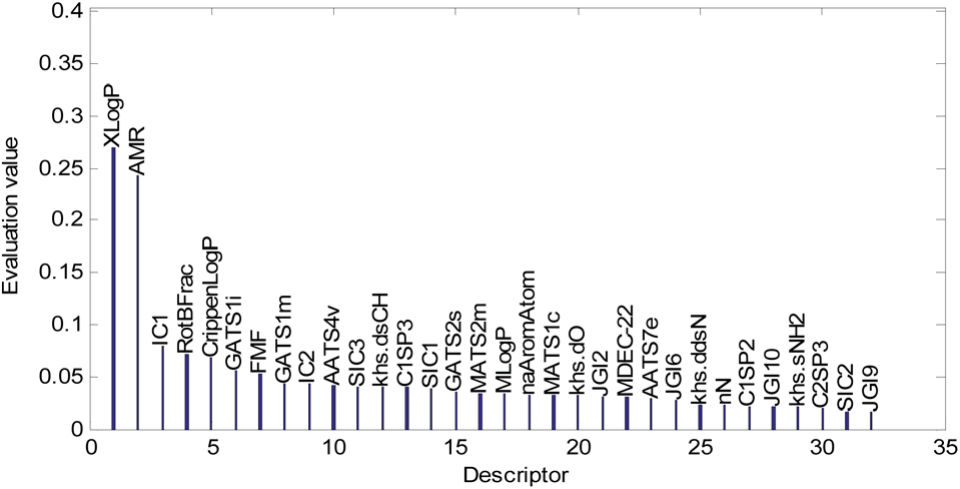
Molecular descriptors selected for QSAR model No. 2.

**Fig. 11:**
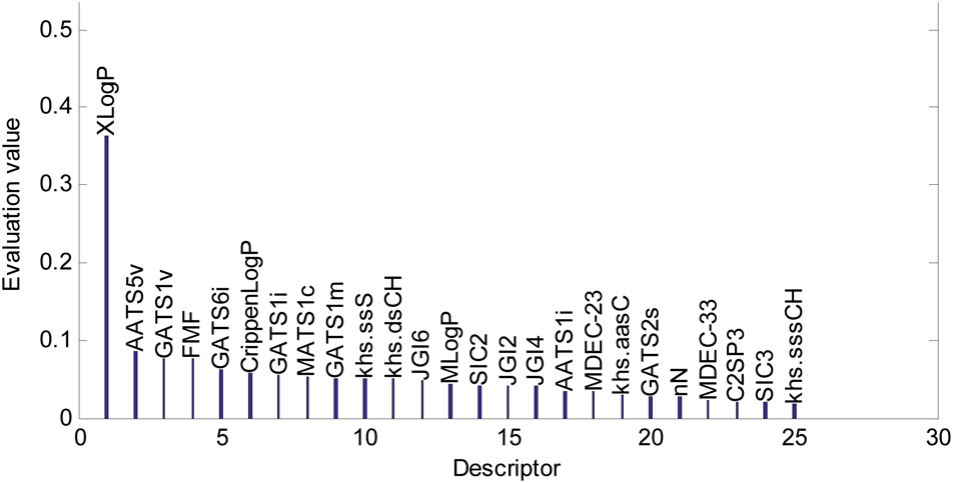
Molecular descriptors selected for QSAR model No. 3.

Descriptors selected for models No. 2 and No. 3 are shown in Fig. 10 and 11, respectively. The descriptors selected for other models are graphically listed in the ESI (Fig. S1–S17).† As can been seen from Fig. 9–11, the descriptors selected for RBF neural networks are different in each model. Meanwhile, in Table 2, we can see that the parameters of the RBF neural networks are also different in each model. This illustrates that, although the HQPSO algorithm cannot guarantee the optimization of the model to the global optimal solution in each run, the robustness and fitting quality of each model are relatively high (in Table 2, the minimum *R*_cv10_^2^ of 20 RBF-based models was greater than 0.7, and their minimum *R*_adj_^2^ was greater than 0.8). Therefore, the joint optimization method adopted in this paper can ensure that the RBF-based model has a good fitting ability and robustness.

In Table 2, we can also find that the external predictive power of the models was unstable, and only 30% of the models had a value of *Q*_ext_^2^ greater than 0.7. However, the QSAR models established by using 0–2D simple descriptors had a higher fitting ability and external predictive power than those of the global model established by using 0–3D descriptors in the literature.^5^ Although *Q*_ext_^2^ was less than 0.7 in other models, the predictive performance was also satisfactory considering the bigger validation set and uncertainties in the modeling data.

### 3.7. Toxicity mechanism interpretation

**Fig. 12:**
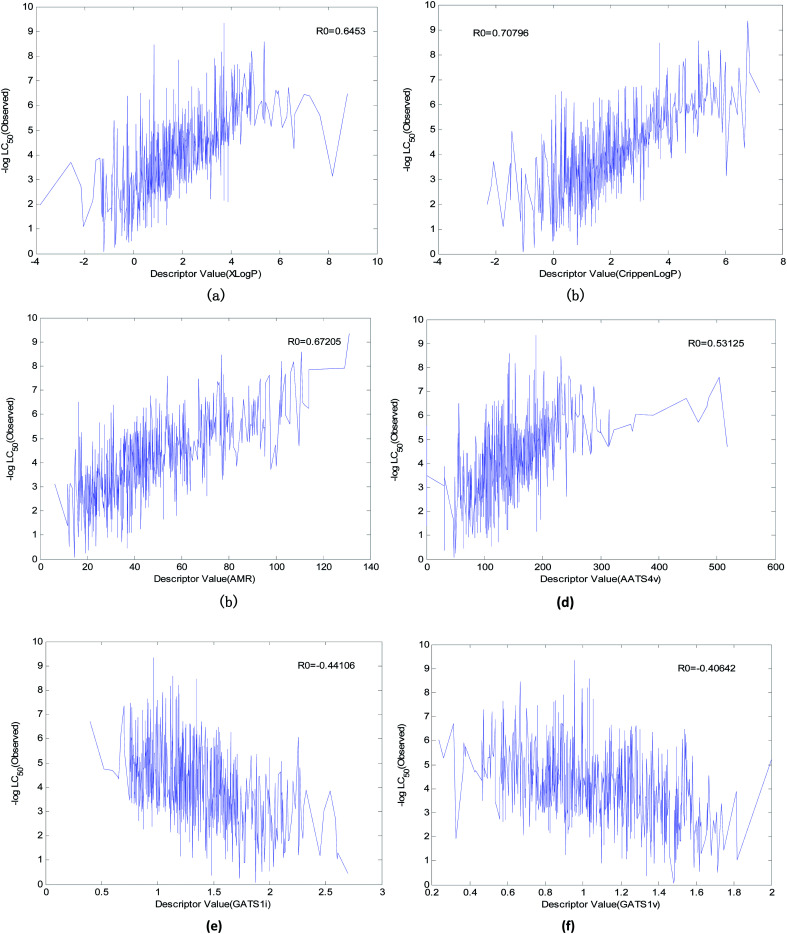
Descriptors with an obvious correlation to acute toxicity. (a) The correlation between Xlog *P* and acute toxicity, (b) correlation between Crippen log *P* and acute toxicity, (c) correlation between AMR and acute toxicity, (d) correlation between AATS4v and acute toxicity, (e) correlation between GATS1i and acute toxicity, and (f) correlation between GATS1v and acute toxicity.

**Fig. 13:**
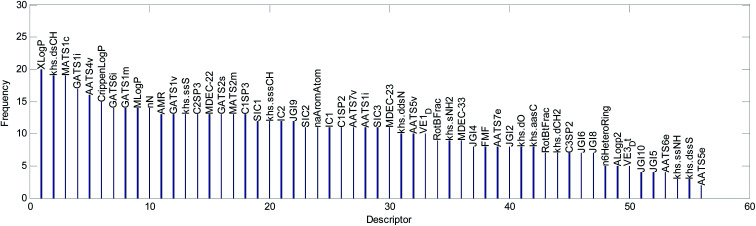
Frequency of the descriptors used in the 20 RBF-based models.

In the OECD principle, it is necessary to analyze the mechanism of the QSAR model. To study the relationships between various descriptors and the acute toxicity of compounds on fathead minnow, we performed the following preparation steps:

(1) We found six descriptors with obvious correlations to acute toxicity through data visualization. The curves between the value of the descriptor and toxicity are shown in Fig. 12.

(2) We counted the frequencies for 56 descriptors used in 20 QSAR models. The statistical results are shown graphically in Fig. 13.

We explained the toxicity mechanisms combined with the times of the descriptors used in the models (Fig. 13), the importance of the descriptors in each model (Fig. 8–10, S1–S17 in the ESI),† and the correlation between the descriptors and acute toxicity (Fig. 12); then, we made the following inferences:

(1) “Xlog *P*” is the most important descriptor affecting acute toxicity. “Xlog *P*” appears in all 20 QSAR models and is the most important descriptor in all models except model No. 5 (“Xlog *P*” is the second most important). “Xlog *P*” is an octanol–water partition coefficient that expresses the lipophilicity of a molecule, and lipophilicity represents the driving force of narcosis toxicity.^1,5^ Molecular toxicity will increase with lipophilicity as a consequence of the enhanced ability of toxicants to enter the organism.^1^ As can be seen from Fig. 12(a), there is a positive correlation between “Xlog *P*” and toxicity, but this is not a simple linear relationship. This relationship illustrates that the acute toxicity of fathead minnow not only results from narcosis toxicity.

(2) “Crippen log *P*” is a water distribution coefficient obtained by another calculation method. It also has an obvious positive correlation with acute toxicity (Fig. 12(b)). Moreover, both the frequency (it appeared 15 times in 20 models) of its occurrence and its importance ranking in the models were relatively high. Therefore, it can also reflect acute toxicity.

(3) “AMR” is molar refractivity. AMR can be used as a measure of electron polarizability in molecules. Previous studies have shown that, for aquatic organisms, the stronger the polarizability, the greater the toxicity of the molecules.^1^ Based on its positive correlation with acute toxicity (Fig. 12(c)), the frequency (it appeared 13 times in 20 models) of its occurrence, and its importance ranking in the models, “AMR” is the same as “Crippen log *P*”. Therefore, AMR is another indicator that reflects acute toxicity.

(4) “AATS4v” is the average Broto–Moreau autocorrelation-lag 4 (weighted by van der Waals volumes), which has an obvious positive correlation with acute toxicity (Fig. 12(d)). “GATS1v” is the Geary autocorrelation-lag 1 (weighted by van der Waals volumes), but it has an obvious negative correlation with acute toxicity (Fig. 12(f)). These two descriptors are related to the van der Waals volumes of molecules. This illustrates that the van der Waals volume of the molecule is not significant when analyzing the acute toxicity of fathead minnow. Although “AATS4v” and “GATS1v” have no clear chemical significance, they can still be used as a reference for the changing trends of acute toxicity.

(5) “GATS1i” is Geary autocorrelation-lag 1 (weighted by first ionization potential), which has an obvious negative correlation with acute toxicity (Fig. 12(e)). This descriptor is related to the first ionization potential. The first ionization potential is the energy required for a gaseous atom in the ground state to lose one electron in its outermost layer. The larger the initial ionization energy, the harder it is for an atom to lose an electron. Thus, the larger the “GATS1i”, the greater the initial ionization energy, and the less likely the compound will both react and produce toxicity. Therefore, “GATS1i” is also one of the indicators that reflects acute toxicity.

(6) The relatively frequent appearance of “khs.dsch”, “MATS1c”, “AATS4v”, “GATS6i”, “GATS1m”, “MlogP”, and “nN” in the models illustrates that these factors may have a certain influence on acute toxicity. However, there is no obvious correlation between them and acute toxicity, so it is difficult to use them to analyze the toxicity mechanism.

(7) The mechanism of the acute toxicity of organic compounds in fathead minnow is very complex and cannot be simply described by using several descriptors. Although the water distribution coefficient, molar refractivity, and first ionization potential are essential factors affecting the acute toxicity of fathead minnow, there is a complex nonlinear relationship between the descriptors and toxicity. Therefore, it is necessary to establish a toxicity prediction model by using a neural network with nonlinear abilities.

## 4. The consensus RBF-based QSAR model

### 4.1. Sub-model selection and consensus modeling

From Table 2, we can see that the external prediction ability of the RBF-based models was unstable (the range of *Q*_ext_^2^ is from 0.6480 to 0.7317). To improve the performance of the established QSAR models, we employed a consensus modeling approach. The idea of consensus modeling is to integrate several weak learners into a strong learner to improve the stability and generalization performance of the QSAR model.^44–46^ To establish a reasonable consensus model, we studied the influence of the several sub-models used in the consensus model. A total of 2–20 RBF-based QSAR models were selected as sub-models to establish a consensus model, and the average output of each sub-model was used as the output of the consensus model.

**Fig. 14:**
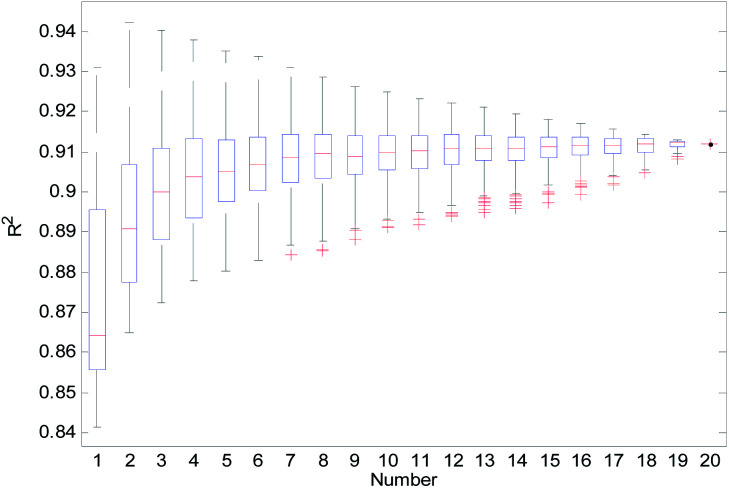
*R* ^2^ statistical results of the consensus models.

**Fig. 15:**
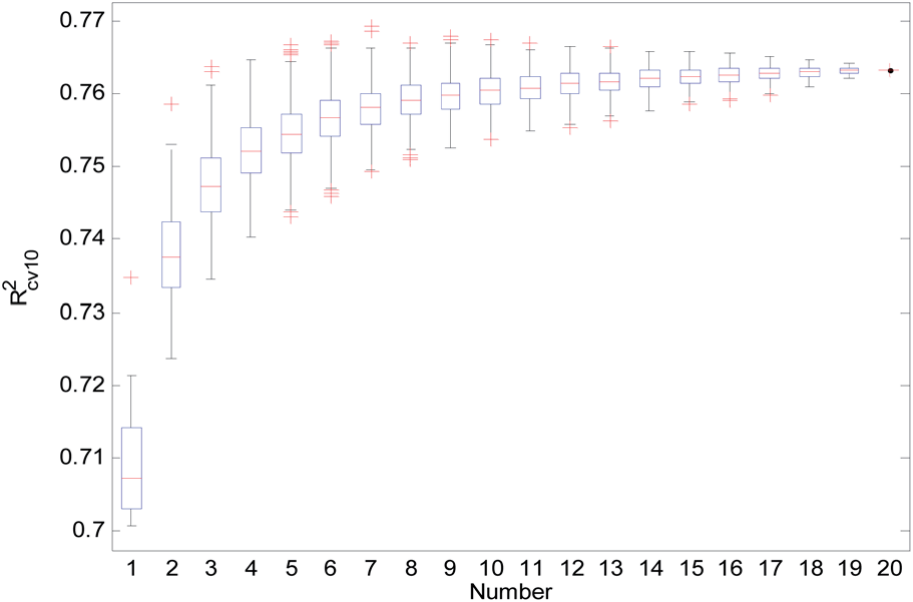
*R*_cv10_^2^ statistical results of the consensus models.

**Fig. 16:**
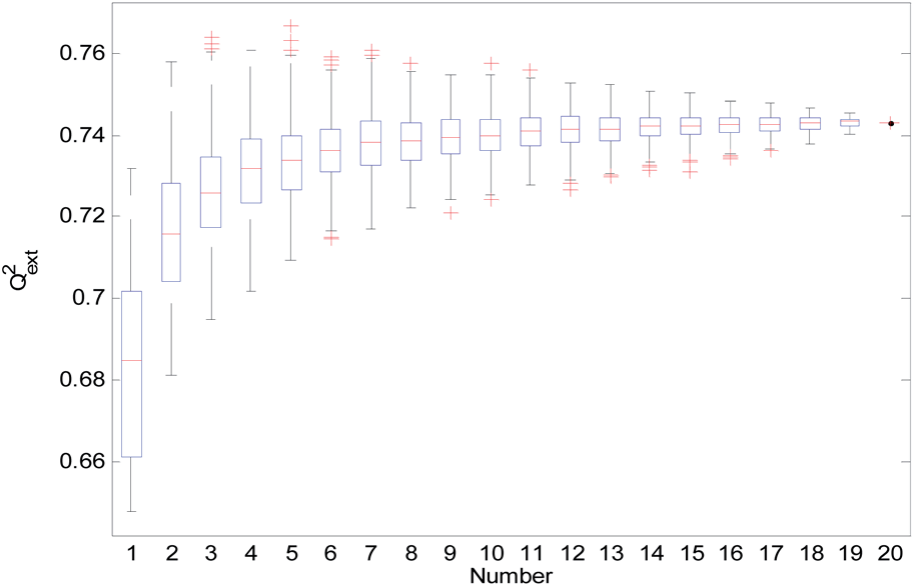
*Q*_ext_^2^ statistical results of the consensus models.

For the consensus models with *N* (*N* = 2, 3, …, 19) sub-models, we established 1000 consensus models with sub-models randomly selected. Then, we calculated the *R*^2^, *R*_cv10_^2^, and *Q*_ext_^2^ of each consensus model. The statistical results of the consensus models with *N* sub-models are shown in the form of boxplots in Fig. 14–16.

In Fig. 14–16, the “Number” on the *X*-axis indicates the number of sub-models used in the consensus model.

When the value of “Number” on the *X*-axis is 1, “*R*^2^”, “*R*_cv10_^2^”, and “*Q*_ext_^2^” on the *Y*-axis represents the statistical results of each RBF-based model (sub-model); when the value of “Number” is 20, “*R*^2^”, “*R*_cv10_^2^”, and “*Q*_ext_^2^” on the *Y*-axis represent the results of the consensus model, including all the 20 sub-models. When the value of “Number” is 2 to 19, “*R*^2^”, “*R*_cv10_^2^”, and “*Q*_ext_^2^” on the *Y*-axis represents the results of the consensus model, including the corresponding number of sub-models.

It can be seen from Fig. 14–16 that the minimum *R*^2^, *R*_cv10_^2^, and *Q*_ext_^2^ of established consensus models increased gradually with an increase of the sub-model, while the maximum *R*^2^, *R*_cv10_^2^, and *Q*_ext_^2^ of established consensus models increased first and then decreased. Meanwhile, the performance of the consensus model also became increasingly more stable with an increase in sub-models. Unlimited increases in the sub-models have little effect in improving the performance of the consensus model.

In the OECD principle, the external validation set cannot participate in the modeling process, so we cannot use the best *Q*_ext_^2^ to choose the sub-models for a consensus model. The sub-models can only be chosen according to the performance of *R*^2^, *R*_cv10_^2^, or the stability of the consensus model. We used 4 methods to select the sub-models, and the comparison results are shown in Table 3.

**Table 3:** Different sub-model selection methods and the external predictive power of the consensus model^a^

Method	No. (sub-models)	*R* ^2^	*R*_cv10_^2^	*Q*_ext_^2^
A	7	0.9139	0.7692	0.7310
B	2	0.9422	0.7396	0.7183
C	6	0.9277	0.7667	0.7271
D	20	0.9118	0.7632	0.7430

a Methods A, B, and C: sub-models were selected according to the maximum *R*_cv10_^2^, maximum *R*^2^, and maximum *R*^2^ × *R*_cv10_^2^ of the consensus models (the maximum *R*_cv10_^2^, *R*^2^, and *R*^2^ × *R*_cv10_^2^ were obtained by searching for the best combination of sub-models through the HQPSO algorithm). D: sub-models were selected according to the stability of the consensus model (the more sub-models, the more stable the consensus model).

From Table 3, we can see that method D has the best *Q*_ext_^2^. Meanwhile, it can be seen from Fig. 16 that the values of *Q*_ext_^2^ are all greater than methods A, B, and C when the number of randomly selected sub-models is greater than 16. As mentioned in studies^38^ and,^47^ a strong internal predictive power does not indicate good external predictive power. A high *R*^2^ and *R*_cv10_^2^ is only a necessary, but not sufficient condition for a QSAR model to have good external predictive power. Our aim was to obtain a stable model with good performance, so we selected method D to establish the consensus model. The performance of this model is as follows: *R*^2^ = 0.9118, *R*_cv10_^2^ = 0.7632 and *Q*_ext_^2^ = 0.7430. The robustness, fitting ability, and external prediction ability of this model are all better than those of RBF-based sub-models.

**Fig. 17:**
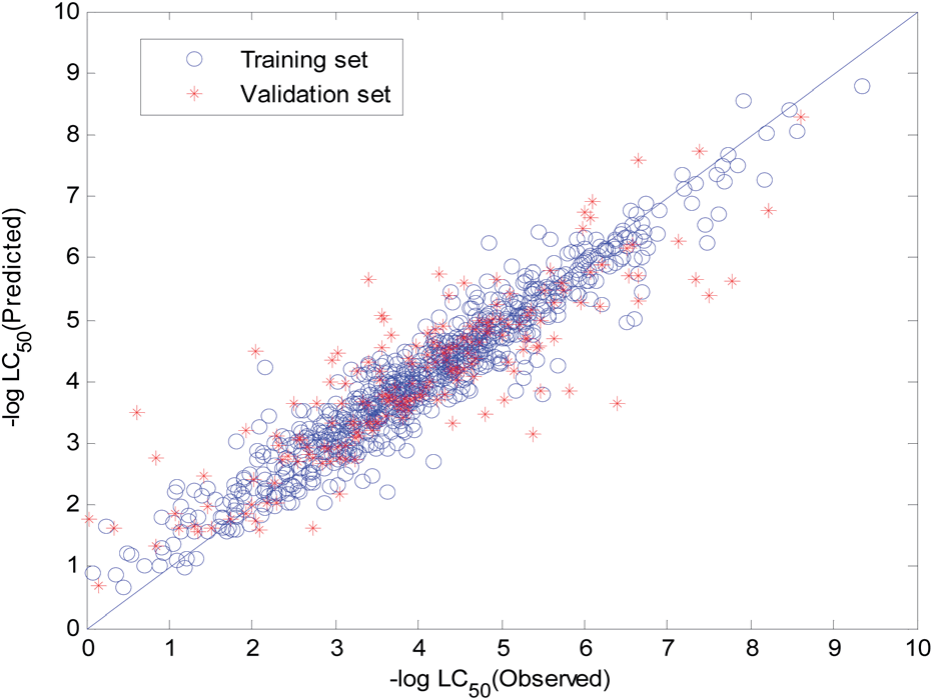
Visual predictive performance of the consensus model.

The visual predictive performance of the consensus model is shown in Fig. 17.

### 4.2. Applicability domain of the consensus model

In the OECD principle, a standard QSAR model must give its AD. The AD indicates the applicable scope of the QSAR model and also indicates the reliability of the prediction results of the QSAR model for newly synthesized compounds.^48–50^ The definition methods for the AD mainly include geometric methods, probability density distribution-based methods, range-based methods, ensemble methods, and chemical similarity distance-based methods.^51,52^ Leverage^53^ is a representative and widely used distance-based AD definition method. It is essentially a method based on the spatial distance information between compounds in the training set, but it does not consider the importance of each descriptor. In the consensus model of this paper, the frequency of each descriptor used in the model is different, which shows that different descriptors have different contribution rates for the output of the model. Therefore, we proposed an FWD-based method to define the AD for the consensus model and compared with the leverage method.

The definition and implementation step of the FWD method are as follows:

Step 1: Calculate the frequency of each descriptor used in the consensus model to form the weight vector *F* = [*f*_1_, *f*_2_, …, *f*_*d*_]. *f*_*j*_ is written in eqn (9):9
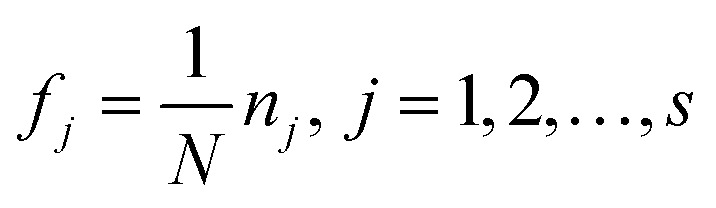
where *f*_*j*_ is the frequency of the *j*th descriptor, and *s* is the number of descriptors used in the consensus model. *N* is the number of sub-models, and *n*_*j*_ is the number of the *j*th descriptor used in the consensus model.

Step 2: The weighted operation for each compound is performed: *v*_*ij*_ = *A*_*ij*_*f*_*j*_. *A*_*ij*_ is the value of the *j*th descriptor of the *i*th compound, and *v*_*ij*_ is the weighted value of the *j*th descriptor of the *i*th compound in the training set.

Step 3: Calculate the center point *C* = [*c*_1_, *c*_2_, …, *c*_*s*_], and *c*_*j*_ is written in eqn (10).10
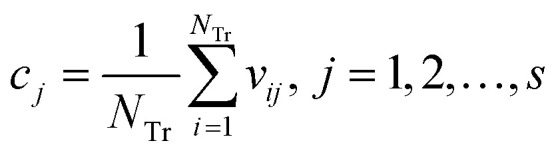
where *N*_Tr_ is the number of compounds in the training set.

Step 4: Calculate the Euclidean distance of each weighted compound to the center *C* using eqn (11).11
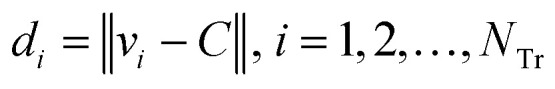


Step 5: Calculate the mean value and standard deviation of *d*. The mean value and standard deviation are expressed as *u* and *δ*, respectively. For the *i*th compound, if *d*_*i*_ > *u* + 3*δ*, we consider the compound outside the AD. Otherwise, it is inside the AD.

**Fig. 18:**
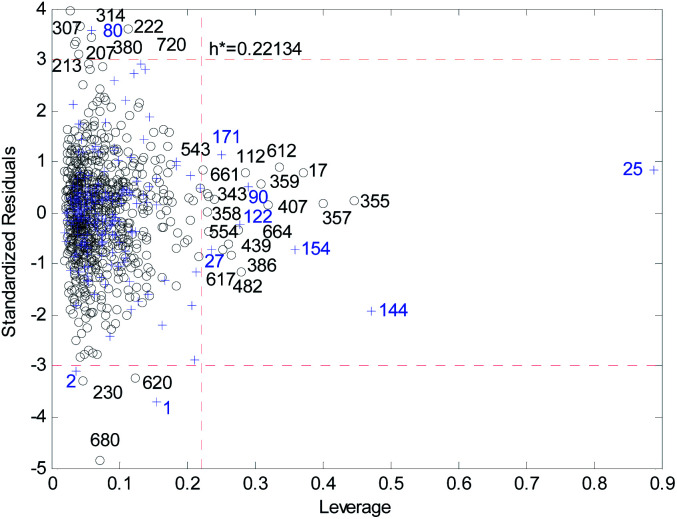
Williams plot of the consensus model based on the leverage method.

**Fig. 19:**
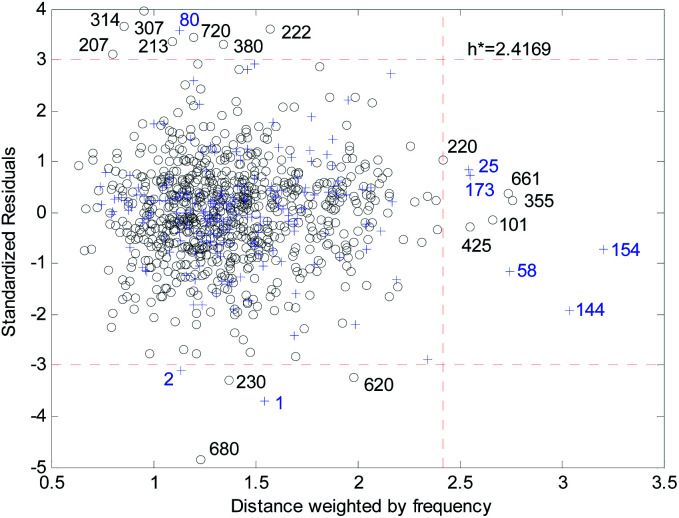
Williams plot of the consensus model based on the FWD method.

Fig. 18 and 19 show the difference between the leverage and FWD methods. In Fig. 18 and 19 black circles represent compounds in the training set, and blue crosses represent compounds in the validation set. In Fig. 18 and 19, the transverse dashed lines represent a ±3 standard residual. In Fig. 18, the vertical dashed line represents a warning leverage = 0.22134. In Fig. 19, the vertical dashed line represents a warning FWD = 2.4169. It can be seen from Fig. 18 and 19 that most compounds in the validation set were predicted within ±3 standardized residuals, which illustrates the good predictive power of the consensus model.

Comparing Fig. 18 with Fig. 19, we can see that, regardless of the AD definition method, the outliers were all in their respective AD. There were more points with good performance outside the AD in Fig. 18, so the AD of the leverage method can be expanded.

In the FWD method, the importance of the descriptors was considered, and the expansion of the AD did not destroy the performance of the consensus model. Therefore, the AD definition method for FWD is also reasonable. According to the FWD method, the AD coverage rate of the consensus model is 99.34% for the training set and 97.45% for the validation set. The application scope of the consensus model is, therefore, wide.

### 4.3. Outlier analysis of the consensus model

**Fig. 20:**
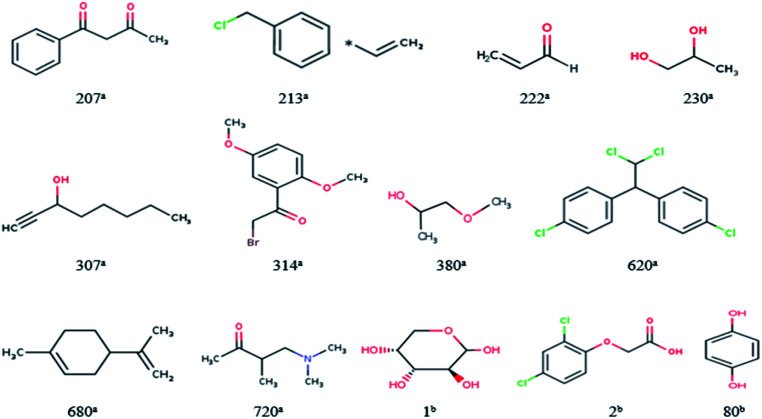
Molecular structures of the outliers. ^a^Outliers in the training set; ^b^outliers in the validation set.

For response variables, 13 compounds were identified as outliers because their standardized residuals were outside the range of ±3 standardized residuals, as shown in Fig. 18 and 19. The molecular structures of the outliers are shown in Fig. 20, and detailed information for these outliers is listed in Table 4.

**Table 4:** Information on the outliers in the consensus model

No.	CAS	Experiment	Predict	Xlog *P*	AMR	AATS4v	GATS1v	GATS1i
207^a^	93-91-4	5.1700	3.8313	1.2900	50.4031	161.0016	0.9795	1.05
213^a^	30 030-25-2	5.69	4.2496	1.8670	40.0635	129.5252	0.8473	0.9904
222^a^	107-02-8	6.52	4.9657	0.2930	16.3660	56.5487	1.1915	1.3333
230^a^	57-55-6	0.24	1.6503	−0.7480	19.1380	49.8783	1.4849	1.7348
307^a^	818-72-4	5.49	3.7873	2.3600	31.2718	108.9875	1.3134	1.3418
314^a^	1204-21-3	6.5900	5.0223	1.6660	61.5298	209.2464	0.9431	1.2976
380^a^	107-98-2	3.6400	2.2181	−0.2290	23.8892	75.6174	1.6168	2.0028
620^a^	72-54-8	4.86	6.2549	3.4030	87.8586	226.7365	0.685	0.9153
680^a^	138-86-3	2.15	4.2357	3.7290	46.0152	106.99	1.25	1.25
720^a^	22104-62-7	4.18	2.7058	0.1610	39.1281	95.6827	1.5601	2.0466
1^b^	87-72-9	0.6	3.5020	−1.4540	29.9609	96.8514	1.1029	1.5242
2^b^	94-75-7	2.0500	4.4881	1.5920	53.1163	222.2119	0.7181	1.1085
80^b^	123-31-9	6.4000	3.6475	0.6540	34.1668	101.8955	0.7759	0.8136

a Outliers in the training set.

b Outliers in the validation set.

Based on the information listed in Table 4 and the correlation between the descriptors and toxicity in Fig. 11, we make the following inferences about the causes of outliers:

For 230^a^ and 1^b^, the values of “Xlog *P*” are small. The values of “AMR” and “AATS4v” are also relatively small, while those of “GATS1v” and “GATS1i” are relatively large. This means that the outliers have low toxicity, which is consistent with their experimental values. The reason for their large errors is that there are few samples close to their toxicity levels in the training set, and the model does not learn their toxicity mechanisms well.

For 620^a^, 680^a^, and 2^b^, the predicted results have large positive errors. 620^a^ and 2^b^ have relatively large values of “AATS4v”, and 620^a^ and 680^a^ have relatively large values of “Xlog *P*”. This may be the reason for the larger prediction results. This also shows that the toxicity mechanisms of these three compounds are complex, which is different to most compounds in the data set.

For 207^a^, 213^a^, 222^a^, 307^a^, 314^a^, 280^a^, 720^a^, and 80^b^, the predicted results have large negative errors. They all have a relatively small water distribution coefficient, which is the most important variable that affects the output of the model, which may be the reason for the small output of the model. This also shows that the acute toxicity of these compounds is determined not only by their lipophilicity but also by other factors. Therefore, to better predict the toxicity of each compound, we need to use a larger number of representative compound structures as a training set to build a more accurate QSAR model.

### 4.4. Comparison of the consensus model with other published models and xgboost-based model

To better illustrate the superiority of the QSAR model established in this paper, we developed a detailed comparative analysis between the model in this paper and the main published models. The results of this comparison can be found in Table 5. Our interest was only on models established with a training set *N*_Tr_ > 700 because using models with small training sets makes it difficult to collect sufficient and diverse molecular information to develop a QSAR model with superior performance and a wide AD.

**Table 5:** Comparison of the current models with previous models (*N*_Tr_ > 700)^a^

Reference	Method	*N*_Tr_	*N*_Va_	*N*_de_	*R* ^2^	*R*_cv_^2^	*Q*_ext_^2^	AD
5	MLR	771	192	8	0.704	0.700	0.641	Yes
15	PNN	800	86	76	0.89–0.99	—	0.52–0.78	No
16	GA-KNN	726	182	6	0.62–0.73	—	0.61–0.77	Yes
Present	RBF-based consensus model	759	196	56	0.9118	0.7632	0.7430	Yes
Present	Xgboost-based model	759	196	56	0.9960	0.7255	0.7235	No

a *N*_Tr_ is the number of compounds in the training set, *N*_Va_ is the number of compounds in the validation set, and *N*_de_ is the number of descriptors used in each model.

As can been seen in Table 5, the MLR,^5^ PNN,^15^ and GA-KNN^16^ algorithms were used to develop models with a training set that *N*_Tr_ > 700. Only the model proposed in this paper and the MLR in^5^ strictly follow the OECD principle. The other algorithms have some disadvantages. PNN and GA-KNN have not been cross-verified; thus, the robustness of these models cannot be guaranteed. PNN does not report AD, which limits its applications. GA-KNN has a poor fitting ability and an unstable external predictive power. Although PNN has a good fitting ability, it cannot illustrate whether the model is over-fitted or not because there is no cross-validation. Meanwhile, its validation set is much smaller than that of the other models, and its external predictive power is unstable.

Under the OECD principle, only MLR can make a fair comparison with the RBF-based consensus model proposed this paper. Compared with MLR, the fitting ability and external predictive power of the RBF-based consensus model have been greatly improved, and the robustness of the model has also been enhanced. Although the number of descriptors used is greater than that of the MLR, this model satisfies the “Topliss ratio” condition, and there is no over-fitting. Meanwhile, the importance of the descriptors and the toxicity mechanisms are explained reasonably.

To further illustrate the superiority of the RBF-based consensus model, we developed a Xgboost-based model with the same training and validation set and compared their performance.

Xgboost is an open source machine learning project developed by Chen and his partners. It has effectively implemented DGBT algorithm and made many improvements in algorithm and engineering.^54^ It has been widely used in Kaggle competition and many other machine learning competitions and achieved good results. It is a very potential machine learning algorithm.

Firstly, the initial parameters range of Xgboost-based model were selected as follows:

learning_rate = [0.01, 0.03, 0.05, 0.07, 0.1]

n_estimators = [700, 900, 1000, 1100, 1300, 1500]

max_depth = [6, 8, 10, 12, 14]

Then, we used grid search method to determine the appropriate parameters of Xgboost.

Finally, when *R*_cv10_^2^ gets the maximum value, the parameters of xgboost algorithm are as follows:

learning_rate = 0.05

n_estimators = 1000

max_depth = 6

The performance of established Xgboost-based model is as follows:

*R* ^2^ = 0.9960, *R*_cv10_^2^ = 0.7255 and *Q*_ext_^2^ = 0.7235.

The detailed information was also listed in Table 5.

Compared with Xgboost, our RBF-based consensus model has better robustness and external prediction ability, even if the fitting performance is worse than Xgboost, it is also satisfactory.

Therefore, according OECD principles, the proposed model in this paper is a reliable model that can be used to predict the acute toxicity of fathead minnow for compounds in an aquatic environment.

## 5. Conclusions

In this study, we developed a fathead minnow acute toxicity model based on an RBF neural network under the OECD principle. A 0–2D descriptor was used to establish a QSAR model to avoid the uncertainty caused by molecular structure optimization when calculating 3D descriptors. The SOM neural network was used to split the dataset to ensure symmetry and fairness of data splitting and generate multiple chemically diverse training and validation sets. The importance evaluation of descriptors and their primary selection based on the mean decrease impurity method was used to simplify the model structure. The HQPSO algorithm with a new fitness function and a new parameter encoding method was employed to jointly optimize the molecular descriptors and parameters of the RBF model to ensure that the most appropriate molecular descriptors were selected under the optimal QSAR model parameters. Combined with frequency and importance of the descriptors used in the RBF-based models, as well as the correlation between the descriptors and acute toxicity, we explained the toxicity mechanism and concluded that the water distribution coefficient, molar refractivity, and first ionization potential are important factors affecting the acute toxicity of fathead minnows. To improve the external prediction ability of the RBF-based models, a consensus model was established, and a new FWD-based AD was defined to illustrate the application scope of the consensus model. The comparison results showed that the model has a wide AD, good fitting ability, good external predictive power, and robustness. The proposed model shows good performance with *R*^2^ = 0.9118, *R*_cv10_^2^ = 0.7632, and *Q*_ext_^2^ = 0.7430 and can act as a reference in the study of aquatic toxicity compounds.

## Conflicts of interest

The authors declare no conflict of interest.

## Supplementary Material

RA-010-D0RA02701D-s001

RA-010-D0RA02701D-s002

RA-010-D0RA02701D-s003

RA-010-D0RA02701D-s004
