# Supplementary material for: A joint optimization QSAR model of fathead minnow acute toxicity based on a radial basis function neural network and its consensus modeling
Source: RSC Adv. 2020 Jun 4;10(36):21292–308. doi: 10.1039/d0ra02701d (PMC9054390; doi:10.1039/d0ra02701d)
Supplement: RA-010-D0RA02701D-s004 [file RA-010-D0RA02701D-s004.pdf]

Electronic Supplementary Material (ESI) for RSC Advances.
This journal is © The Royal Society of Chemistry 2020

Supplementary Materials

A Joint Optimization QSAR Model of Fathead Minnow Acute Toxicity Based on a Radial Basis Function Neural Network and Its Consensus Modeling

[a,b]Yukun Wang [1,2] and [b*]Xuebo Chen

[a] School of Chemical Engineering, University of Science and Technology Liaoning, No. 185, Qianshan, Anshan 114051, Liaoning, China; wyk410@163.com

[b] School of Electronic and Information Engineering, University of Science and Technology Liaoning, No. 185, Qianshan, Anshan 114051, Liaoning, China

* Correspondence: xuebochen@126.com; Tel.: +864125928367

Table S1 Deleted compounds and the reason for deletion

No.	CAS	Reason for deletion
1	71862-02-7	*
2	98434-34-5	*
3	10-15-1	*
4	3944-76-1	*
5	22037-97-4	*
6	56348-39-1	*
7	61096-84-2	*
8	108-20-3	**

* We can't query its SMILES file on the website (https://chem.nlm.nih.gov/ chemidplus/chemidlite.jsp), so we can't compute its descriptors . The CAS code may be wrong. ** We cannot calculate its descriptors using PaDEL software.

Table S2 Details of 56 selected descriptors

NO.	Descriptor name	Type	Description	Extended Class
1	XLogP	XLogPDescriptor	Prediction of logP based on the atom-type method called XLogP.	Molecular property descriptors
2	khs.dsCH	ElectrotopologicalStateAtomType Descriptor	Count of atom-type E-State: =CH-	E-state
3	MATS1c	Moran AutocorrelationDescriptor	Moran autocorrelation - lag 1 / weighted by charges	Autocorrelation descriptors
4	GATS1i	Geary AutocorrelationDescriptor	Geary autocorrelation - lag 1 / weighted by first ionization potential	Autocorrelation descriptors
5	GATS4v	Geary AutocorrelationDescriptor	Geary autocorrelation - lag 4 / weighted by van der Waals volumes	Autocorrelation descriptors
6	CrippenLogP	CrippenDescriptor	Crippen's LogP	Constitutional descriptors
7	GATS6i	Geary AutocorrelationDescriptor	Geary autocorrelation - lag 6 / weighted by first ionization potential	Autocorrelation descriptors
8	GATS1m	Geary AutocorrelationDescriptor	Geary autocorrelation - lag 1 / weighted by mass	Autocorrelation descriptors
9	MLogP	MannholdLogPDescriptor	Mannhold LogP	Molecular property descriptors
10	nN	AtomCountDescriptor	Number of nitrogen atoms	Constitutional descriptors
11	AMR	AMRDescriptor	Molar refractivity	Constitutional descriptors
12	GATS1v	Geary AutocorrelationDescriptor	Geary autocorrelation - lag 1 / weighted by van der Waals volumes	Autocorrelation descriptors
13	Khs.ssS	ElectrotopologicalStateAtomType Descriptor	Count of atom-type E-State: -S-	E-state descriptors
14	C2SP3	CarbonTypesDescriptor	Singly bound carbon bound to two other carbons	Topological descriptors
15	MDEC-22	MDEDescriptor	Molecular distance edge between all secondary carbons	Topological descriptors
16	GATS2s	Geary AutocorrelationDescriptor	Geary autocorrelation - lag 2 / weighted by I-state	Autocorrelation descriptors
17	MATS2m	Moran AutocorrelationDescriptor	Moran autocorrelation - lag 2 / weighted by mass	Autocorrelation descriptors

18	C1SP3	CarbonTypesDescriptor	Singly bound carbon bound to one other carbon	Topological descriptors
19	SIC1	StructuralInformationContentDescriptor	Structural information content index (neighborhood symmetry of 1-order)	Basak descriptors
20	khs.sssCH	ElectrotopologicalStateAtomTypeDescriptor	Count of atom-type E-State: >CH-	E-state descriptors
21	IC2	InformationContentDescriptor	Information content index (neighborhood symmetry of 2-order)	Basak descriptors
22	JGI9	TopologicalChargeDescriptor	Mean topological charge index of order 9	Topological descriptors
23	SIC2	StructuralInformationContentDescriptor	Structural information content index (neighborhood symmetry of 2-order)	Basak descriptors
24	naAromAtom	AromaticAtomsCountDescriptor	Number of aromatic atoms	Constitutional descriptors
25	IC1	InformationContentDescriptor	Information content index (neighborhood symmetry of 1-order)	Basak descriptors
26	C1SP2	CarbonTypesDescriptor	Doubly bound carbon bound to one other carbon	Topological descriptors
27	AATS7v	Average Broto-Moreau AutocorrelationDescriptor	Average Broto-Moreau autocorrelation - lag 7 / weighted by van der Waals volumes	Autocorrelation descriptors
28	AATS1i	Average Broto-Moreau AutocorrelationDescriptor	Average Broto-Moreau autocorrelation - lag 1 / weighted by first ionization potential	Autocorrelation descriptors
29	SIC3	StructuralInformationContentDescriptor	Structural information content index (neighborhood symmetry of 3-order)	Basak descriptors
30	MDEC-23	MDEDescriptor	Molecular distance edge between all secondary and tertiary carbons	Topological descriptors
31	khs.ddsN	ElectrotopologicalStateAtomTypeDescriptor	Count of atom-type E-State: -N<<	E-state descriptors
32	AATS5v	Average Broto-Moreau AutocorrelationDescriptor	Average Broto-Moreau autocorrelation - lag 5 / weighted by van der Waals volumes	Autocorrelation descriptors
33	VE1D	BaryszMatrixDescriptor	Coefficient sum of the last eigenvector from Barysz matrix / weighted by van der Waals volumes	Topological descriptors
34	RotBFrac	RotatableBondsCountDescriptor	Fraction of rotatable bonds, excluding terminal bonds	Constitutional descriptors
35	Khs.sNH2	ElectrotopologicalStateAtomTypeDescriptor	Count of atom-type E-State: -NH2	E-state descriptors
36	MDEC-33	MDEDescriptor	Molecular distance edge between all tertiary carbons	Topological descriptors
37	JGI4	TopologicalChargeDescriptor	Mean topological charge index of order 4	Topological descriptors
38	FMF	FMFDescriptor	Complexity of a molecule	Topological descriptors
39	AATS7e	Average Broto-Moreau	Average Broto-Moreau	Autocorrelation

		AutocorrelationDescriptor	autocorrelation - lag 7 / weighted by Sanderson electronegativities	descriptors
40	JGI2	TopologicalChargeDescriptor	Mean topological charge index of order 2	Topological descriptors
41	khs.dO	ElectrotopologicalStateAtomType Descriptor	Count of atom-type E-State: =O	E-state descriptors
42	khs.aasC	ElectrotopologicalStateAtomType Descriptor	Count of atom-type E-State: :C:-	E-state descriptors
43	RotBtFrac	RotatableBondsCountDescriptor	Fraction of rotatable bonds, including terminal bonds	Constitutional descriptors
44	khs.dCH2	ElectrotopologicalStateAtomType Descriptor	Count of atom-type H E-State: =CH2	E-state descriptors
45	C3SP2	CarbonTypesDescriptor	Doubly bound carbon bound to three other carbons	Topological descriptors
46	JGI6	TopologicalChargeDescriptor	Mean topological charge index of order 6	Topological descriptors
47	JGI8	TopologicalChargeDescriptor	Mean topological charge index of order 8	Topological descriptors
48	n6HeteroRing	RingCountDescriptor	Number of 6-membered rings containing heteroatoms (N, O, P, S, or halogens)	Constitutional descriptors
49	ALogP2	ALOGPDescriptor	Square of ALogP	Constitutional descriptors
59	VE3Dt	DetourMatrixDescriptor	Logarithmic coefficient sum of the last eigenvector from detour matrix	Topological descriptors
51	JGI10	TopologicalChargeDescriptor	Mean topological charge index of order 10	Topological descriptors
52	JGI5	TopologicalChargeDescriptor	Mean topological charge index of order 5	Topological descriptors
53	AATS6e	Average Broto-Moreau AutocorrelationDescriptor	Average Broto-Moreau autocorrelation - lag 6 / weighted by Sanderson electronegativities	Autocorrelation descriptors
54	khs.ssNH	ElectrotopologicalStateAtomType Descriptor	Count of atom-type E-State: -NH-	E-state descriptors
55	khs.dssS	ElectrotopologicalStateAtomType Descriptor	Count of atom-type E-State: >S=	E-state descriptors
56	AATS5e	Average Broto-Moreau AutocorrelationDescriptor	Average Broto-Moreau autocorrelation - lag 5 / weighted by Sanderson electronegativities	Autocorrelation descriptors

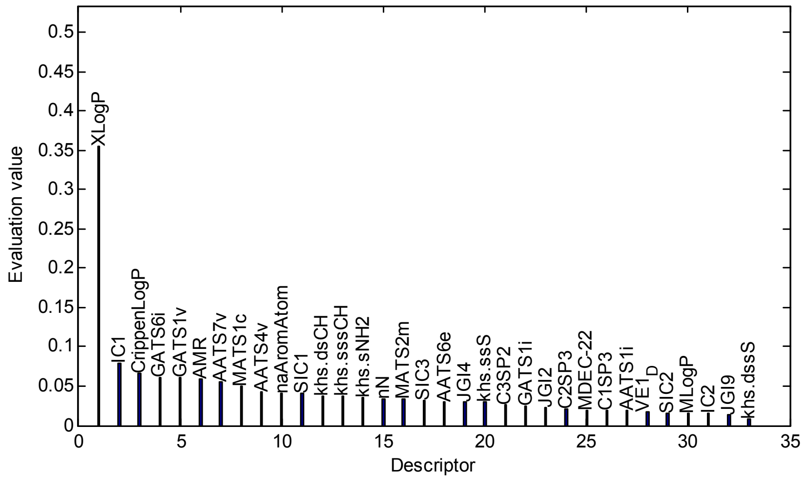

Fig. S1 Molecular descriptors selected for the BRF-based model No.4

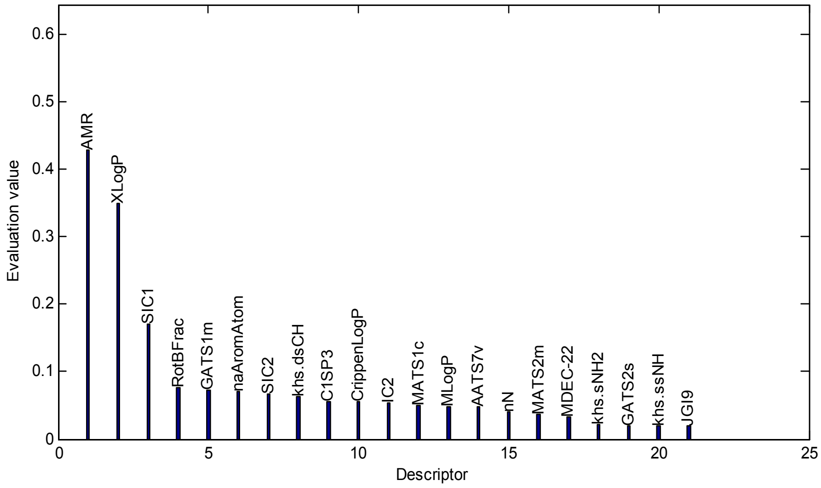

Fig.S2 Molecular descriptors selected for the RBF-based model No.5

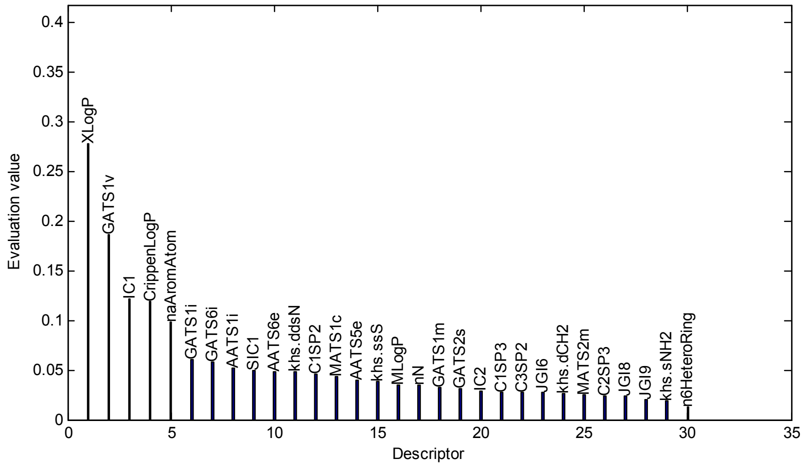

Fig. S3 Molecular descriptors selected for the RBF-based model No.6

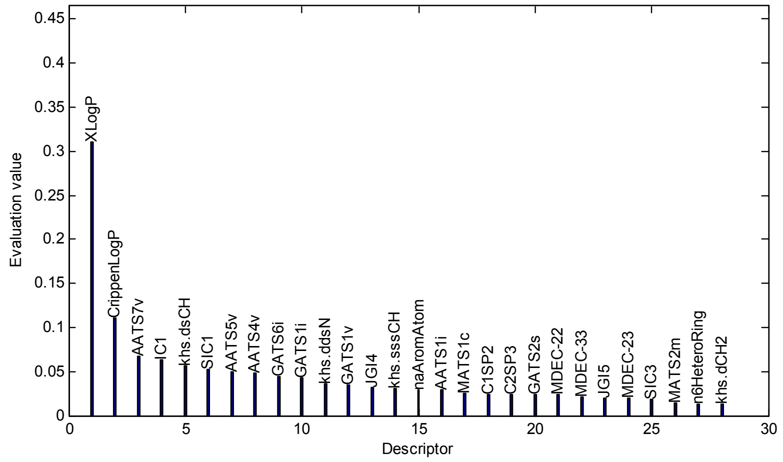

Fig. S4 Molecular descriptors selected for the RBF-based model No.7

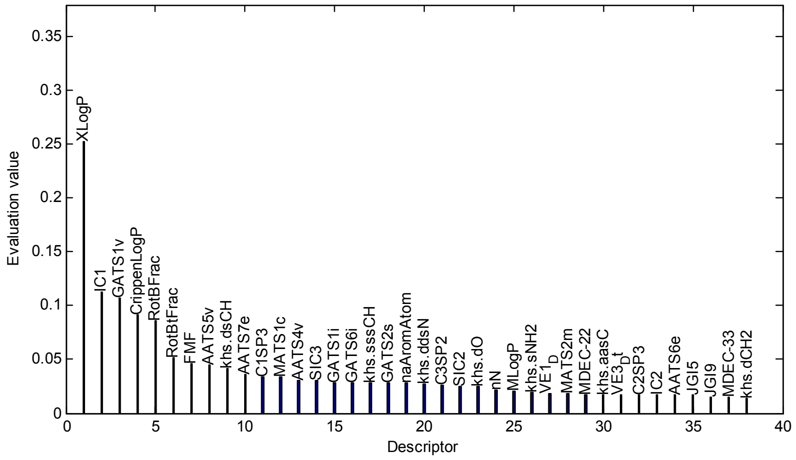

Fig.S5 Molecular descriptors selected for the RBF-based model No.8

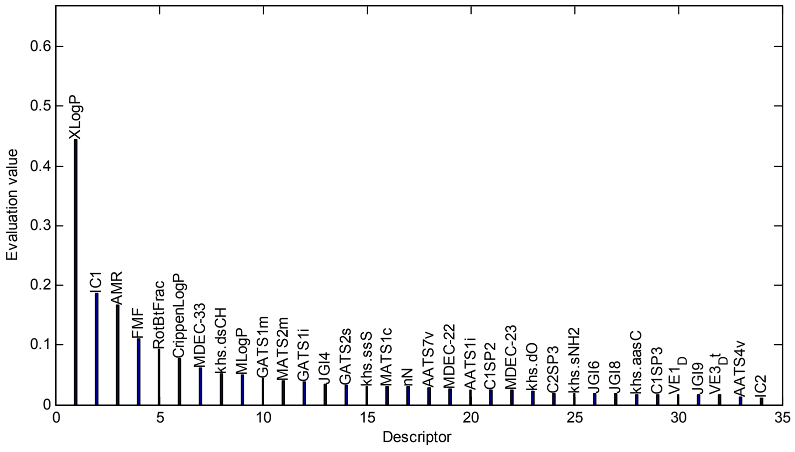

Fig. S6 Molecular descriptors selected for the RBF-based model No.9

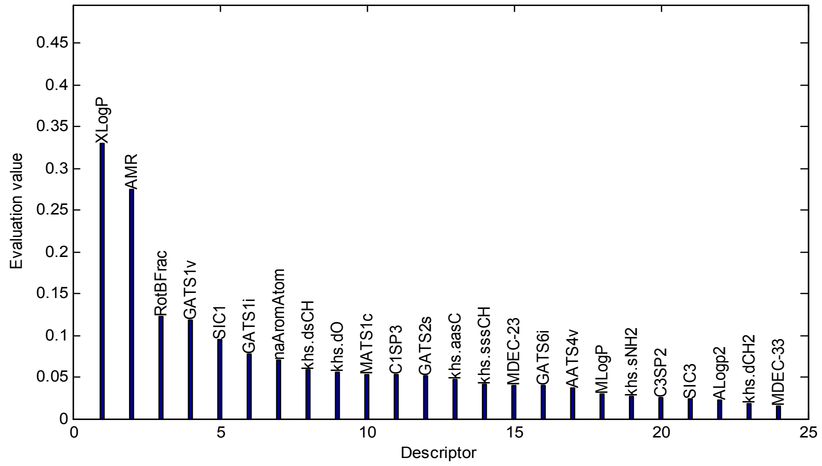

Fig. S7 Molecular descriptors selected for the RBF-based model No.10

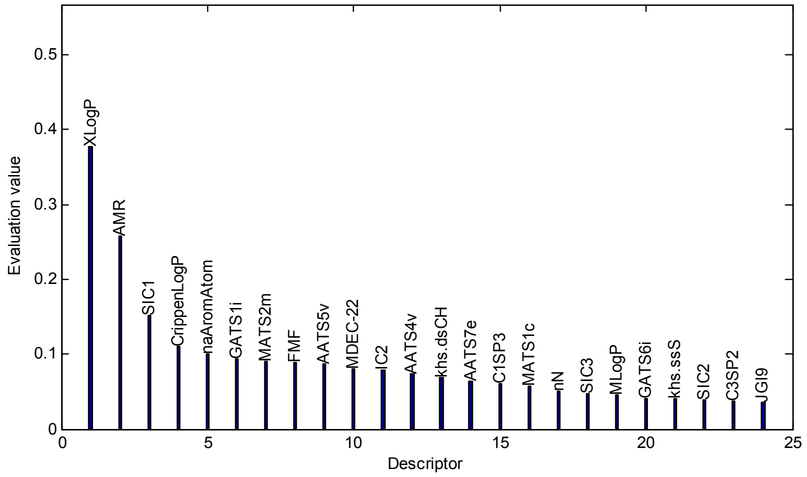

Fig. S8 Molecular descriptors selected for the RBF-based model No.11

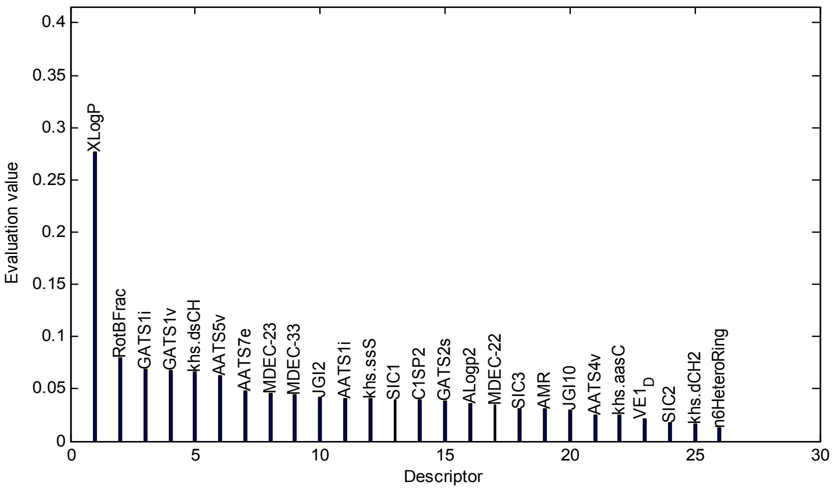

Fig.S9 Molecular descriptors selected for the RBF-based model No.12

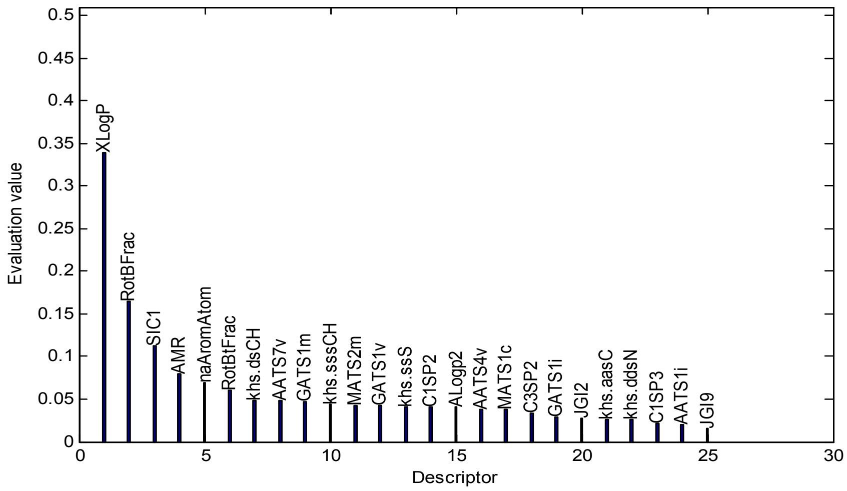

Fig. S10 Molecular descriptors selected for the RBF-based model No.13

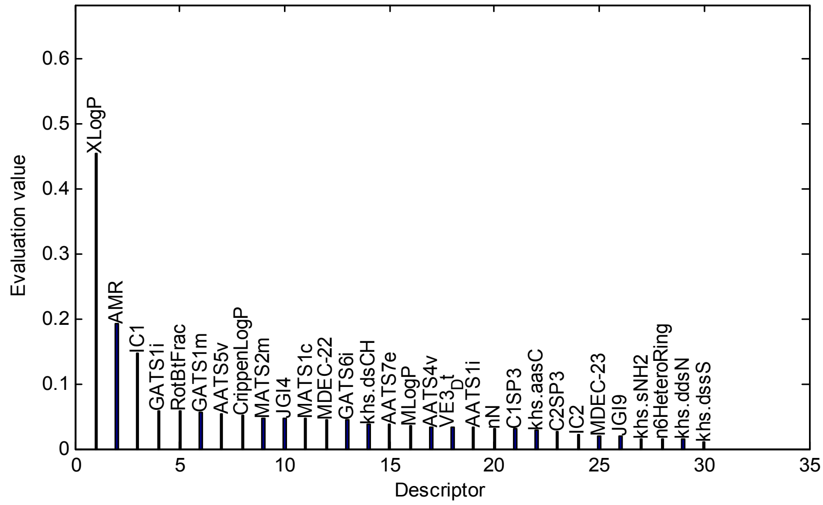

Fig. S11 Molecular descriptors selected for the RBF-based model No.14

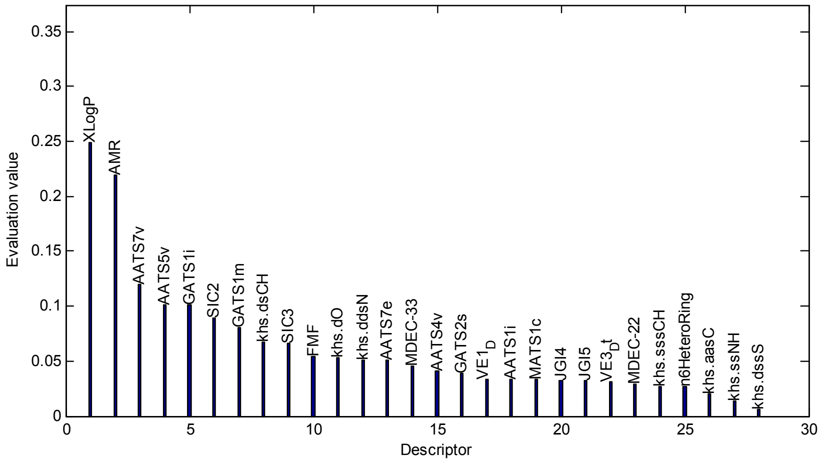

Fig. S12 Molecular descriptors selected for the RBF-based model No.15

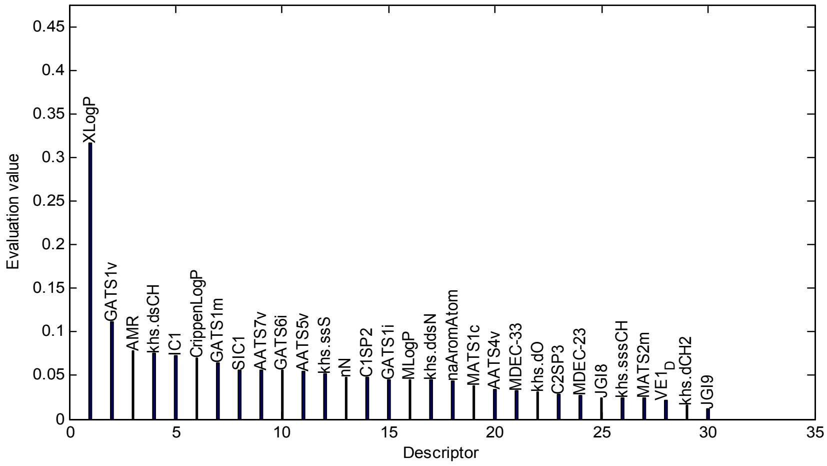

Fig. S13 Molecular descriptors selected for the RBF-based model No.16

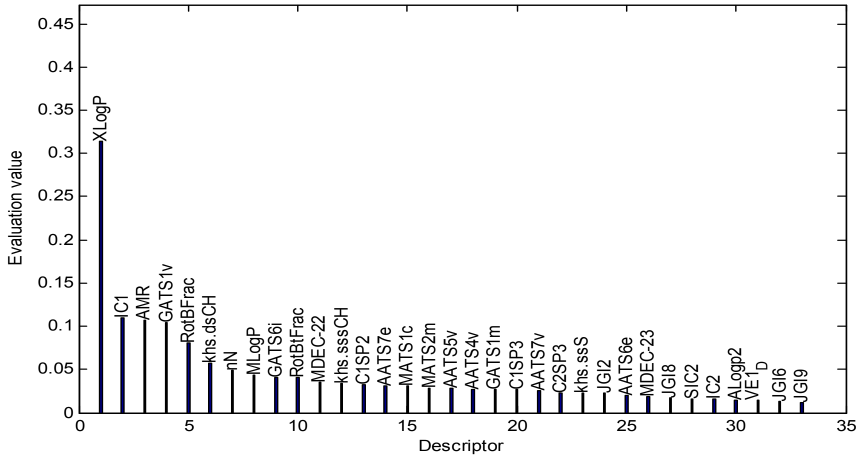

Fig. S14 Molecular descriptors selected for the RBF-based model No.17

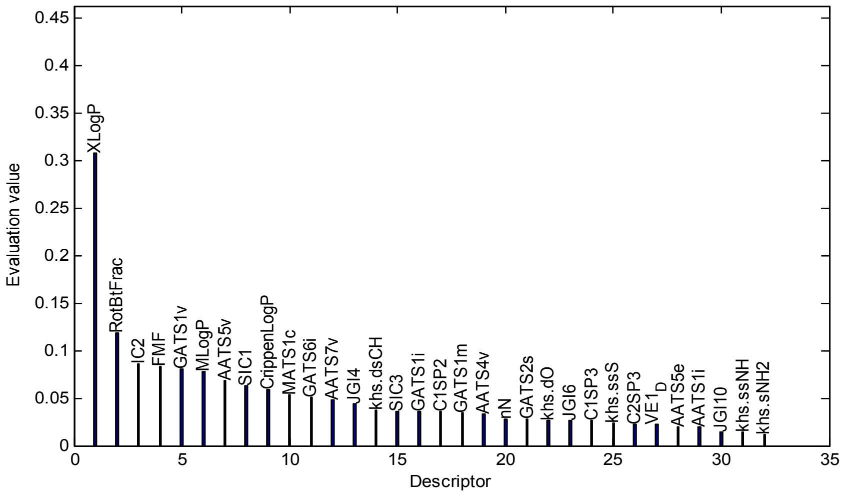

Fig.S15 Molecular descriptors selected for the RBF-based model No.18

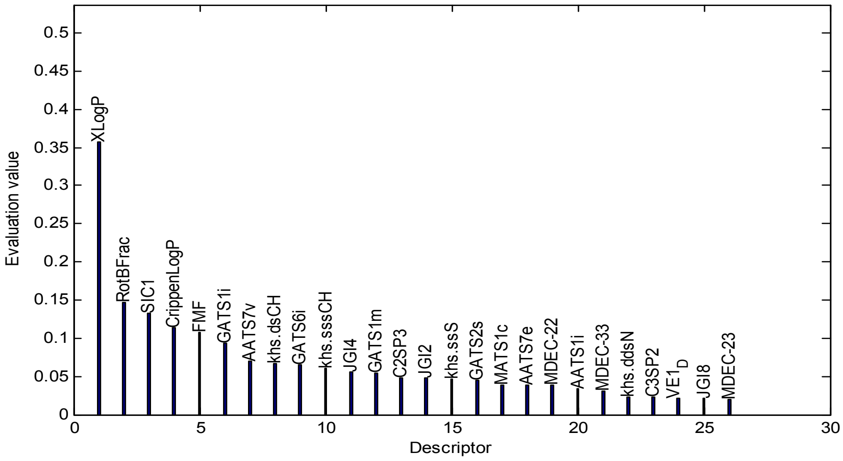

Fig. S16 Molecular descriptors selected for the RBF-based model No.19

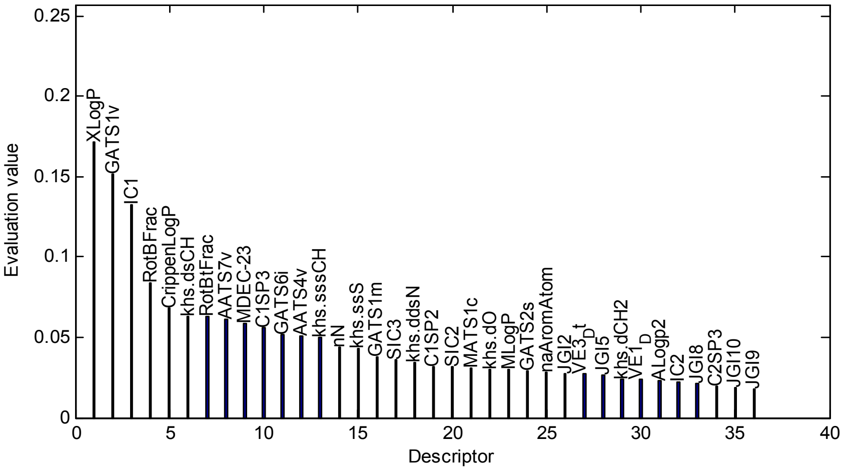

Fig. S17 Molecular descriptors selected for the RBF-based model No.20
